# Ciliary GPCR‐based transcriptome as a key regulator of cilia length control

**DOI:** 10.1096/fba.2021-00029

**Published:** 2021-07-05

**Authors:** Yuki Kobayashi, Sakura Tomoshige, Kosuke Imakado, Yuko Sekino, Noriko Koganezawa, Tomoaki Shirao, Giovanne B. Diniz, Tatsuo Miyamoto, Yumiko Saito

**Affiliations:** ^1^ Graduate School of Integrated Sciences for Life Hiroshima University Hiroshima Japan; ^2^ Endowed Laboratory of Human Cell‐Based Drug Discovery Graduate School of Pharmaceutical Sciences The University of Tokyo Tokyo Japan; ^3^ Department of Neurobiology and Behavior Graduate School of Medicine Gunma University Maebashi Japan; ^4^ AlzMed, Inc. Tokyo Japan; ^5^ California National Primate Research Center University of California Davis CA USA; ^6^ Department of Genetics and Cell Biology Research Institute for Radiation Biology and Medicine Hiroshima University Hiroshima Japan

**Keywords:** G protein‐coupled receptor, melanin‐concentrating hormone, PDLIM5, primary cilia, transcriptome

## Abstract

The primary cilium is a plasma membrane‐protruding sensory organelle that efficiently conveys signaling cascades in a highly ordered microenvironment. Its signaling is mediated, in part, by a limited set of GPCRs preferentially enriched in the cilium membrane. This includes melanin‐concentrating hormone (MCH) receptor 1 (MCHR1), which plays a role in feeding and mood. In addition to its receptor composition, the length of the cilium is a characteristic parameter that is implicated in its function. We previously found that MCH can dynamically shorten cilia length via the Gi/o and Akt pathways in both MCHR1‐expressing hTERT‐RPE1 cells (hRPE1 cells) and rat hippocampal neurons. However, the detailed mechanisms by which MCH regulates cilia length through ciliary MCHR1 remains unclear. In this study, we aimed to determine the transcriptome changes in MCHR1‐expressing hRPE1 cells in response to MCH to identify the target molecules involved in cilia length regulation via MCHR1 activation. RNA sequencing analysis of ciliated cells subjected to MCH treatment showed upregulation of 424 genes and downregulation of 112 genes compared with static control cells. Validation by quantitative real‐time PCR, knocking down, and CRISPR/Cas9‐mediated knockout technology identified a molecule, PDZ and LIM domain‐containing protein 5 (PDLIM5). Thus, it was considered as the most significant key factor for MCHR1‐mediated shortening of cilia length. Additional analyses revealed that the actin‐binding protein alpha‐actinin 1/4 is a crucial downstream target of the PDLIM5 signaling pathway that exerts an effect on MCHR1‐induced cilia shortening. In the endogenous MCHR1‐expressing hippocampus, transcriptional upregulation of PDLIM5 and actinin 1/4, following the application of MCH, was detected when the MCHR1‐positive cilia were shortened. Together, our transcriptome study based on ciliary MCHR1 function uncovered a novel and important regulatory step underlying cilia length control. These results will potentially serve as a basis for understanding the mechanism underlying the development of obesity and mood disorders.

## 1 INTRODUCTION

Primary cilia are solitary nonmotile microtubule‐based organelles found in multiple mammalian cell types, projecting from the apical surface into the surrounding environment.^1^, ^2^ These organelles are important regulators of the cell cycle, both structurally––through the anchorage of the mother centriole and cilium disassembly at cell cycle entry––and molecularly through the organization of multiple developmental relevant signaling pathways, including sonic hedgehog (Hh) and wingless/int (Wnt).^2^, ^3^ In addition to their role in cell proliferation, primary cilia are important structures to sense the cellular microenvironment, propagating signals into juxtaposed cytoplasmic structures.^4^ To achieve that, the primary cilium is enriched in proteins involved in signal transduction, including specialized components such as platelet‐derived growth factor receptor α, transforming growth factor β receptor, and a limited set of G protein‐coupled receptors (GPCRs).^5^, ^6^, ^7^, ^8^, ^9^ This allows primary cilia to respond to selective physiological ligands and produce cilia‐initiated cellular processes.

While primary cilia are considered nonmotile structures due to absence of beating as observed in multiciliated cells, they are dynamic structures nonetheless, with assembly/disassembly and length, both playing a predominant role in their function.^10^, ^11^, ^12^, ^13^ Ciliary synthesis and maintenance are achieved by a microtubule‐based transport system consisting of intraflagellar transport (IFT) and other motor proteins that carry cargo proteins along the microtubular axoneme.^1^, ^14^ In addition to specialized transport, cilium‐destined proteins are sorted by a specialized protein complex known as the Bardet–Biedl Syndrome complex (BBSome), which links cilia‐destined proteins to the IFT machinery.^1^, ^15^ These molecular complexes work in tandem to ensure that the ciliary membrane and cilioplasm are biochemically distinct from the plasma membrane and cytoplasm, creating a privileged compartment that is capable of selective signal transduction.^2^, ^5^, ^6^, ^7^, ^8^, ^9^ The disruption of these systems can cause a large number of overlapping and frequently syndromic human diseases collectively known as ciliopathies.^16^

In addition to ciliopathies, atypical ciliary morphology has been reported for different animal and cellular models of neurological disorders that are not typically considered ciliopathies.^17^, ^18^, ^19^, ^20^ Mounting evidence, however, suggests ciliary length is also relevant in physiological conditions, as spatially restricted ciliary shortening is observed in obese BBS4‐, leptin‐, and leptin receptor‐deficient mice.^21^, ^22^ Therefore, studies on cilia length regulation might reveal essential determinants of energy homeostasis linked to obesity in individuals with ciliopathies, as well as other neuronal functions at the cellular level.^23^, ^24^ Physiological modulation of ciliary length is achieved through multiple soluble factors, including leptin, lysophosphatidic acid, prostaglandin, and lithium.^25^, ^26^, ^27^, ^28^, ^29^ However, there is no unified cilia length‐determining pathway, as multiple signaling components that alter cilia length regardless of cell cycle period have been identified. Prostaglandin E, for example, activates the ciliary EP4 receptor, resulting in cyclic AMP‐mediated signaling cascade that increases the anterograde velocity of IFT in hRPE1 cells,^25^ while leptin elongates primary cilia through PTEN‐GSK3β signaling, recruiting the transcription of IFT genes and actin cytoskeletal destabilization.^26^ Of note, early studies demonstrated that pharmacological perturbation of actin polymerization increases cilia length in addition to cilia formation, suggesting an antagonistic relationship between polymerization of the actin cytoskeleton/F‐actin branching and cilia assembly.^30^, ^31^, ^32^ So far, extensive studies using cilia‐specific cell biological and molecular approaches have identified multiple actin regulatory factors that alter cilia length.^32^, ^33^, ^34^, ^35^, ^36^ The investigation of individual cilia‐regulating signaling network, therefore, is necessary to fully elucidate this complex biological process.

Melanin‐concentrating hormone (MCH), an orexigenic neuropeptide predominantly synthesized in the lateral hypothalamus of vertebrates, has emerged as a major regulator of ciliary length.^37^, ^38^, ^39^ Neurons that synthesize MCH project throughout the brain, where released MCH acts through MCH receptor 1 (MCHR1), a GPCR that acts as the sole receptor of MCH in rodents.^40^, ^41^ The MCH‐MCHR1 system constitutes a powerful regulatory system associated with food intake, energy expenditure, mood, and the sleep–wake cycle.^38^ Rodent MCHR1 is preferentially located in the primary cilia of neurons in the olfactory tubercle, hypothalamus, accumbens, and hippocampal formation, with MCHR1 in the latter concentrated in CA1 and CA3 regions but not in the dentate gyrus.^42^, ^43^, ^44^ We have previously described that MCH‐MCHR1 binding mediates cilia length shortening in four different cultures of ciliated cells, including human retinal pigmented epithelial RPE1 cells (hRPE1) exogenously expressing MCHR1, rat hippocampal dissociated and slice neurons, and human‐induced pluripotent stem cell‐derived cortical neurons.^44^, ^45^, ^46^ We also demonstrated that Gi/o‐dependent Akt phosphorylation is a major contributor of the initial stage of MCH‐induced primary cilia shortening, leading to a downstream depolymerization of cytoplasmic tubulin and actin polymerization.^44^, ^45^, ^47^ However, the critical intermediate events underlying Gi/o‐Akt‐dependent MCHR1‐initiated cilia shortening along with cytoskeletal rearrangement remain unclear. Therefore, in the present study we aimed to identify the genes responsible for ciliary length regulation via the MCH‐ciliary MCHR1 axis.

By applying RNA sequencing (RNA‐seq) coupled with quantitative RT‐PCR (qRT‐PCR) analyses, siRNA‐mediated knockdown, and CRISPR/Cas9‐mediated gene targeting, we identified PDZ and LIM domain‐containing protein 5 (PDLIM5) as the most significant components associated with MCHR1‐induced shortening of cilia length. We further demonstrated the crucial importance of the F‐actin network regulator alpha‐actinin 1/4 as a downstream target of PDLIM5. This is the first study to analyze ciliary GPCR‐based transcriptome profiles in terms of ciliary dynamics, revealing major intermediaries in the MCH‐MCHR1 signaling cascade. Our approach also serves as a template for future studies aiming to identify key insights into the signaling role of other ciliary GPCRs.

## 2 MATERIALS AND METHODS

### 2.1 hRPE1 cell culture

hRPE1 cell clone stably expressing MCHR1:EGFP and SSTR3 cells stably expressing SSTR3:EGFP were established as described previously.^45^ The cells were grown in Dulbecco's modified Eagle's medium/F12 culture medium (Sigma‐Aldrich) supplemented with 10% fetal bovine serum, 0.5 mM sodium pyruvate, 15 mM HEPES (pH 7.5), 10 µg/ml hygromycin B, and 1% penicillin G/streptomycin at 37°C under 5% CO_2_. To induce ciliogenesis in confluent cultures, the complete medium was replaced with serum‐free medium, and the cells were cultured for a further 24 h.

### 2.2 Generation of SSTR3 stable expression hRPE1 cell line

hRPE1 cells stably expressing SSTR3:EGFP were established with a lentiviral vector. pRRLsinPPT‐SSTR3‐EGFP‐IRES‐Neo was transfected into HEK293FT cells using Polyethylenimine Max (Polysciences Inc.) together with the packaging plasmids (pRSV‐REV, pMD2.g, and pMDL/pRRE). The culture medium was replaced at 8 h after transfection. Culture media containing the lentiviral vector were collected at 24, 36, and 48 h after transfection, filtered through a 0.45‐mm filter (Sartorius), and centrifuged at 32,000 *g* at 4°C for 4 h using an R15A rotor and a Himac CR22G centrifuge (Hitachi Koki Co. Ltd.). The precipitated viral vector was resuspended in Opti‐MEM. hRPE1 cells stably expressing SSTR3:EGFP (SSTR3 clone) were established by transduction with the lentiviral vector and selection with 400 µg/ml of G418 (Nacalai Tesque). As heterogeneous cell populations were observed during cell passages of the original stable cells, 12 individual clones were selected and seeded on Lab‐Tek 8‐well plates. Clones were chosen using a mean cilia length of 3–6 µm as an index under a fluorescence microscope (BZ‐9000; Keyence). The clones were further tested for the efficiency of SST14‐induced cilia shortening by culture in serum‐free medium containing SST14 (Peptide Institute) for 6 h. Finally, one clone, named SSTR3 clone, was selected to analyze the time‐ and dose dependency, effects on cilia length of pharmacological compounds.

### 2.3 Effect of drug treatment on hormone‐induced cilia length control

The following drugs were used in this study: pertussis toxin (PTX; List Biological Laboratories Inc.); AG1478 (Cell Signaling Technology); ZCL278, EHT1864 (Cayman Chemicals); SP600125, Y27632 (Wako); NSC23766, SB203580, and Akti1/2 (Abcam). MCHR1:EGFP and SSTR3:EGFP clone cells were serum‐starved for 24 h prior to the pretreatment. Cells were pretreated with 120 ng/ml PTX for 24 h in serum‐starved medium. For other reagents, the final concentrations of the reagents and the pretreatment periods were: 10 µM AG1478 (30 min); 10 µM Y27632 (30 min); 50 µM ZCL278 (30 min); 10 µM EHT1864 (30 min); 3 µM NSC23766 (30 min); 30 µM SB203580 (30 min), 3 µM SP600125 (30 min), and 3 µM Akti1/2 (30 min).

### 2.4 Protein extraction and western blotting

Western blotting analyses were performed as described previously.^45^ To generate whole‐cell extracts, hRPE1 cells were washed with phosphate‐buffered saline (PBS), and lysed with ice‐cold sodium dodecyl sulfate (SDS) sample buffer (50 mM Tris‐HCl pH 6.8, 2% SDS, 50 mM β‐mercaptoethanol, 10% glycerol). The lysates were homogenized by sonication (Sonicator Ultrasonic Processor W‐225; Wakenyaku Ltd.) at 4°C using five 30 s bursts at 20% power. The proteins were separated by SDS–PAGE and electrotransferred to Hybond‐P PVDF membranes (GE Healthcare UK Ltd.). After blocking with 5% skim milk, the membranes were probed with anti‐phospho‐JNK (#9251; Cell Signaling Technology; 1:1000), anti‐JNK (sc‐474; Santa Cruz Biotechnology; 1:1000), anti‐phospho‐Akt (Ser473) (#9271; Cell Signaling Technology; 1:1000), anti‐phospho‐Akt (Thr308) (#9275; Cell Signaling Technology; 1:1000), anti‐Akt (#9272; Cell Signaling Technology; 1:2000), anti‐PDLIM5 (sc‐515621; Santa Cruz Biotechnology; 1:1000), anti‐glyceraldehyde 3‐phosphate dehydrogenase (GAPDH) (016–25523; Wako; 1:8000), anti‐alpha‐actin (MAB1501; Sigma‐Aldrich; 1:5000), anti‐alpha‐tubulin (NB600‐506SS; Novus; 1:12000), or anti‐alpha‐actinin (D6F6; Cell Signaling Technology; 1:1000) antibody. The bound antibodies were detected with a horseradish peroxidase‐conjugated goat anti‐mouse, anti‐rabbit, or anti‐rat IgG secondary antibody (NA931, NA934; GE Healthcare, 7077; Cell Signaling Technology). The reactive bands were visualized with a Pierce Western Blotting Substrate (Thermo Fisher Scientific). To evaluate the involvement of Gi/o activity, the transfected cells were pretreated with 120 ng/ml PTX for 18 h. Rac‐1 inhibitor NSC23766 was treated with 10 µM for 30 min. Band intensity was analyzed and expressed as the relative intensity by ImageJ software (National Institutes of Health).

### 2.5 WES system

To generate whole‐cell extracts, MCHR1:EGFP clone cells were washed with PBS, and lysed with ice‐cold sample buffer (20 mM Tris‐HCl [pH 7.5], 150 mM NaCl, 0.5% TX‐100, 50 mM Tris‐HCl pH 6.8). The lysate was passed 15 times through a 21‐gauge needle fitted to 1‐ml syringe, centrifuged at 15,000 rpm for 15 min, and take the supernatant as the protein sample. Protein levels of PDLIM5 and GAPDH were quantified using an automated capillary‐based western blotting system, a device “WES” from ProteinSimple. All steps were performed with the manufacturer's reagents according to the user manual. Briefly, 4 µl (0.8 µg) of cell lysates were mixed with 1 µl of 5x fluorescent master mix contained dithiothreitol and heated at 95°C for 5 min. The prepared cell lysates, primary and secondary antibodies, a biotinylated ladder, and HRP chemiluminescent substrate were dispensed into designated wells in a 384‐well‐assay plate. Separation, stacking, and immobilization were automatically performed using separation matrix for high‐molecular weight proteins (Standard pack 3, 66–440 kDa, ProteinSimple) and for low‐molecular weight proteins (Standard pack 1, 12–230 kDa, ProteinSimple). The data were analyzed with compatible Compass software according to the ProteinSimple protocols. Primary antibodies used were anti‐PDLIM5 mouse monoclonal antibody (sc‐515621; Santa Cruz Biotechnology) and anti‐GAPDH mouse monoclonal antibody (sc‐32233; Santa Cruz Biotechnology) at the dilution rate of 1:50 and 1:100, respectively.

### 2.6 RNA‐seq and RNA‐seq data analysis

Differences in gene expression were investigated between untreated and treated MCHR1:EGFP clone cells with 1 µM MCH for 2 h. For RNA sequencing, total RNA of each sample was isolated with the ReliaPrep RNA Cell Miniprep System and treated with DNase I to eliminate genomic DNA following manufacturer's instructions (Promega). The RNA concentration was examined using a NanoDrop spectrophotometer (Thermo Fisher Scientific). RNA samples with RNA Integrity Number (RIN) ≥9.5, as determined by the Agilent 2100 Bioanalyzer (Agilent Technologies), were used for cDNA library preparation.

The cDNA library construction, sequencing, and transcriptome resequencing analysis were performed by Macrogen. Briefly, the cDNA libraries were prepared TruSeq RNA Sample Prep Kit v2. Sequencing was performed using the Illumina HiSeq platform that generates paired end reads of 101 bp. To assess sequencing read quality, FastQC v0.10.0 (Babraham Bioinformatics) was run on the Illumina FastQ input files. The raw reads were trimmed to remove the adapter sequence, the specific sequence of the other Illumina, and the reads less than 36 bp using the Trimmomatic ver 0.32. The control sample produced 64.62 million (64,620,710) reads, and total read bases were 6.5 G bp. The GC content was 50.09% and Q30 was 94.72%. For the MCH 2 h sample, 64.70 million (64,696,368) reads were produced with a total read base of 6.5 G bp. The GC content was 49.92% and Q30 was 95.31%. Trimmed reads were mapped to human reference genome (UCSC hg19) with TopHat version 2.0.13, splice‐aware aligner. The total number of mapped reads for each transcript was determined and normalized to detect the number of fragments per kilobase of exon per million fragments mapped (FPKM) using Cufflinks. Among the analyzed samples, genes with FPKM values of zero in ≥1 of the samples were excluded. For differentially expressed genes (DEG) identification, the values of log2 (FPKM + 1) were calculated and normalized by quantile normalization (15937 genes, 2 samples). The results of DEG analysis showed 536 genes which satisfied |fc| ≥ 2 conditions in comparison pair.

### 2.7 Quantitative RT‐PCR (qRT‐PCR)

Total RNA was extracted from each group of cells using a ReliaPrep RNA Cell Miniprep System (Promega) according to the manufacturer's instructions. The quantity and quality of the extracted RNA were assessed by a NanoDrop spectrophotometer (Thermo Fisher Scientific). RNA was reverse‐transcribed into cDNA using a PrimeScript RT Reagent Kit with gDNA Eraser (Perfect Real Time; Takara) primed with RT Primer Mix (Oligo dT Primer and Random 6 mers) in accordance with the manufacturer's instructions.

The sequences of target genes, together with that of GAPDH as a reference gene, were obtained from GenBank. Quantitative PCR was performed using SYBR Green and specific primers designed for amplification of the target genes using Primer‐BLAST (https://www.ncbi.nlm.nih.gov/tools/primer‐blast) and synthesized by FASMAC. Primer sequences are listed in Table S1. All amplifications were carried out in triplicate. qRT‐PCR was performed in a 10 µl reaction containing 5 µl of Brilliant III Ultra‐Fast SYBR Green QPCR Master Mix (Agilent Technologies), 10 ng of total input RNA, and 2 pmol primer set. The qRT‐PCR protocol was 95°C for 30 s, followed by 40 cycles of 95°C for 15 s and 60°C for 1 min in an ABI PRISM 7000 system (Applied Biosystems). Evaluation of the housekeeping gene GAPDH allowed normalization of the expression levels of the target genes to the amount of input cDNA. ΔCT between GAPDH and each target gene (CT_target gene_ − CT_reference gene_) was calculated for the case and control groups, and ΔΔCT was calculated by subtracting the mean ΔCT of the case samples from the mean ΔCT of the control samples. Fold change expression was evaluated by the 2^−ΔΔCT^ method.

### 2.8 RNA interference

For siRNA, 5 × 10^4^ MCHR1:EGFP clone cells in 4‐well Labtech plates or 8 × 10^4^ cells in 12‐well plates were seeded, cultured for 18 h, and transfected with 20 pmol siRNA per 5 × 10^4^ cells using Lipofectamine RNAiMAX (Thermo Fisher Scientific). Transfected cells were cultured in normal growth medium for 18 h and then serum‐starved for 24 h before analysis. Stealth RNAi siRNA oligonucleotides (Thermo Fisher Scientific) were negative control med GC scramble (12935‐300), siRGS3#1 (HSS184285), siPDLIM5#1 (HSS173779), siPDLIM5#2 (HSS173778), siAlpha‐actinin 1#2 (HSS189573), and siAlpha‐actinin 4 #1 (HSS100124). Quantitation of ciliation frequencies showed no differences between the scramble‐ and each siRNA‐transfected cell (Table S2). In addition, we designed rescue experiments involving PDLIM5 genes containing silent mutations in the siRNA target regions. The PDLIM5 rescue vector was prepared by using the pcDNA3.1‐based PDLIM5 expression vector as a template and replacing nucleotide fragments (gBlocks, Integrated DNA Technologies) containing degenerate mutations with an infusion method (Takara) (Figure S1A). In the siRNA rescue experiment, MCHR1:EGFP clone cells were treated with PDLIM5 siRNA#1 and #2 together, and then the rescue HA‐PDLIM5 vector or mock vector was transiently transfected with jetOPTIMUS (Polyplus Transfection). After that, the effect of MCH (1 µM: 6 h) was evaluated by targeting cells in which HA‐PDLIM5 was detected by cell immunostaining.

### 2.9 Generation of knockout cell lines

Knockout cells were generated by the CRISPR/ObLiGaRe (obligate ligation––gated recombination) method.^48^ For the construction of an expression vector of both sgRNA targeting gene and spCas9, a pair of annealed oligodeoxynucleotides designed on the target sites addressed in the key resource table with overhangs of the BbsI restriction enzyme site were inserted into the *pX330‐U6‐Chimeric_BB‐CBh‐hSpCas9* plasmid (#42230; Addgene). The targeting plasmid vector consisted of a BbsI restriction enzyme site flanked with a CMV promoter‐driven *hsvTK‐2A‐Neo* cassette as described previously in the pBluescript SK II^+^ backbone.^49^ The CRISPR/Cas9 system for the target site (Table S3) was ligated into the targeting vector backbone mediated by the BbsI restriction enzyme site. Successful integration of oligodeoxynucleotides into each plasmid vector was verified by Sanger sequencing.

A total of 2 × 10^5^ hRPE1 cells were seeded into one well of a six‐well plate 24 h before lipofection. Then, 20 ng of the targeting vector and 600 ng of the *pX330* plasmid vector for the target gene editing were transfected into the cells using Lipofectamine LTX (Thermo Fisher Scientific), in accordance with the manufacturer's protocol. At 48 h after the transfection, the transfected cells were reseeded into 15‐cm dishes and then subjected to selection using 2 mg/ml G418. Eight to sixteen drug‐resistant cell colonies were then picked up on days 14–18 after transfection. These colonies were divided into two aliquots: One was transferred into a well of a 96‐well plate for clonal expansion, while the other was lysed and used for PCR and direct‐sequence genotyping. As described previously,^48^ PCR genotyping to screen the hRPE1 cell clones was performed using extracted genomic DNA as a template and KOD‐FX Neo DNA polymerase (Toyobo) with three types of primer pair: the first primer pair for detecting the target gene locus (Table S3), the second primer pair consisting of the forward primer in the target gene locus and *Neo^r^*‐reverse primer (5′‐GCGGATCTGACGGTTCACTAAACCA‐GC‐3′) for detecting the forward insertion of the drug‐resistant gene cassette into the target gene locus, and the third primer pair consisting of the reverse primer in the target gene locus and *Neo^r^*‐reverse primer for detecting the reversed insertion. PCR products were run on 2.0% agarose gel. The wild‐type‐sized PCR products amplified with the third primer pair were directly sequenced to determine the presence or absence of insertion or deletion mutations using 3130 Genetic Analyzer (Applied Biosystems) (Table S4). For rescue experiments, PDLIM5^−/−^ hTERT‐RPE1 cells were co‐transfected with HA‐tagged PDLIM5 and MCHR1:EGFP by jetOPTIMUS. The cells co‐expressed PDLIM and MCHR1 were further tested for the efficiency of MCH‐induced cilia shortening by culture in serum‐free medium containing MCH for 6 h.

### 2.10 Ciliary length measurement using a fluorescence microscope

The cells grown on Lab‐Tek plates or coverslips were fixed with 3.7% formaldehyde in PBS for 15 min. After two washes with PBS, the cells were permeabilized with 0.1% Triton X‐100, transferred into a blocking solution (20% goat serum in PBS) for 30 min, and incubated with mouse anti‐acetylated tubulin (Ac‐tub) (T7451; Sigma‐Aldrich; 1:2000) and rabbit anti‐GFP (598; MBL; 1:2000) primary antibodies for 16–24 h at 4°C. The bound antibodies were detected using appropriate secondary antibodies (Alexa Fluor 546‐conjugated goat anti‐mouse IgG or Alexa Fluor 488‐conjugated goat anti‐rabbit IgG; Life Technologies Co.).^45^ Because primary cilium in vitro cell culture is usually lying flat along the coverslip, the length from a two‐dimensional image was measured using a line measurement tool PhotoRuler Ver. 1.1 software (The Genus Inocybe, Hyogo, Japan) under the BZ‐9000 fluorescence microscope (Keyence). Data for at least 100 cilia per treatment were obtained from at least three independent experiments, and the values are presented as means ± SEM.

### 2.11 Acquisition of high‐resolution photographs using a confocal microscope

The MCHR1:EGFP clone cells and SSTR3:EGFP clone cells were plated (8000 cells/cm^2^) on coverslips (12 mm; Matsunami Glass) in 24‐well plates. The cells were fixed with fresh 4% formaldehyde in PBS for 10 min at room temperature and subsequently fixed with 20°C methanol for 10 min. After two washes with PBS, the cells were permeabilized with 0.1% Triton X‐100 for 5 min, transferred into a blocking solution (1% BSA in PBS) for 20 min, and incubated with mouse anti‐AcTub (T7451; Sigma‐Aldrich; 1:2000), mouse anti‐PDLIM5 (sc‐515621; Santa Cruz Biotechnology; 1:1000), and rabbit anti‐alpha‐actinin (D6F6; Cell Signaling Technology; 1:1000) primary antibodies for 24 h at 4°C, in Can Get Signal immunostain B (Toyobo). After washing with PBS, the cells were incubated with appropriate species‐specific secondary antibodies, comprising Alexa Fluor 546‐conjugated goat anti‐mouse IgG (Thermo Fisher Scientific; 1:300), Alexa Fluor 647‐conjugated goat anti‐mouse IgG (Thermo Fisher Scientific; 1:300), Alexa Fluor 546‐conjugated goat anti‐rabbit IgG (Thermo Fisher Scientific; 1:300), and in Can Get Signal immunostain B for 3 h. If necessary, actin was stained with phalloidin‐iFluor 647 (ab176759; Abcam; 1:400) for 1 h. Finally, the cells were washed, counterstained with 4′,6‐diamidino‐2‐phenylindole (DAPI; 1 µg/ml) for 10 min at room temperature, washed again, and mounted on glass slides with VECTOR Shield (Vector Laboratories). SSTR3 clones were stained with rabbit anti‐Cdk5Rap2 (06‐1398; Merck Millipore, 1:200) primary antibody and Alexa Fluor 647‐conjugated goat anti‐rabbit IgG (Invitrogen, 1:300) secondary antibody according to Section 2.10. High‐magnification images were obtained with an FV3000 confocal microscope equipped with a 60× oil‐immersion objective (Olympus).

### 2.12 Animals

Male Wistar rats (Charles River Laboratories Japan) (*n* = 4) and male C57B/6J mice (Charles River Laboratories Japan) (*n* = 8) were maintained under a 12‐h/12‐h light/dark cycle (lights on at 08:00 h) in a temperature‐ and humidity‐controlled holding room. Food and water were available ad libitum. Rat primary dissociated hippocampal cultures were performed according to the guidelines of the Animal Care and Experimentation Committee (Gunma University, Showa Campus, Maebashi, Japan) and conformed to the NIH guidelines for the use of animals in research. Regarding protocols for fasting were reviewed by the Hiroshima University Animal Care Committee and met the Japanese Experimental Animal Research Association standards, as defined in the Guidelines for Animal Experiments (1987).

### 2.13 Rat primary dissociated hippocampal cultures

The hippocampal cells prepared from Wistar rats at embryonic day 18 were dissociated by trypsin treatment and stored in liquid nitrogen until the day of use.^44^, ^50^ For culturing, frozen hippocampal cells (now commercially available as SKY Neuron from AlzMed, Inc.) were thawed in a thermostat bath at 37°C for 3 min, then transferred to 50 ml tube. Minimum essential medium (Invitrogen) supplemented with 10% fetal bovine serum, 0.6% glucose, and 10 nM pyruvic acid was transferred dropwise to the 50‐ml tube containing hippocampal cells. The neurons were plated (5000 cells/cm^2^) on coverslips (18 mm; Matsunami Glass) coated with poly‐L‐lysine (1 mg/ml) in 24‐well plates and incubated at 37°C in a 5% CO_2_ incubator. After 1 h, the medium was changed to primary‐GM (Neurobasal medium with B‐27 supplement, GlutaMAX1, and penicillin G/streptomycin). After 4 days in culture, 1.5 µM cytosine arabinoside diluted with primary‐GM was added to remove glial cells. One‐third of the primary‐GM was changed every week. On days 18 of culture (18 DIV), the cells were treated with MCH and collected for RNA isolation. After RNA extraction, it was performed according to Section 2.7.

Immunohistochemical staining was performed according to previous studies.^44^ Cells were fixed with Histochoice fix solution for 10 min, and blocked with 5% donkey serum and 0.1% Triton X‐100 in PBS for 45 min. The cells were incubated with primary antibodies overnight at 4°C. For immunostaining, rabbit anti‐AC3 (RPCA‐ACIII; Encor Biotechnology; 1:5000) and goat anti MCHR1 C‐17 (sc‐5534; Santa Cruz Biotechnology; 1:300) primary antibodies were used. The cells were incubated with appropriate species‐specific secondary antibodies, comprising Alexa Fluor 488‐conjugated donkey anti‐rabbit IgG (Thermo Fisher Scientific; 1:400) and Alexa Fluor 546‐conjugated donkey anti‐goat IgG (Thermo Fisher Scientific; 1:400) for 90 min. Finally, the cells were counterstained with DAPI and mounted on glass slides with VECTOR Shield. High‐magnification images were obtained with an FV3000 confocal microscope equipped with a 60× oil‐immersion objective (Olympus).

### 2.14 Fasting of mice

Eight‐week‐old male mice were fasted for 48 h with free access to water,^44^ and investigated for target genes mRNA expression levels. Mice fasted for 48 h exhibited approximately 20% decrease in body weight compared with control mice. The mice were anesthetized by isoflurane and euthanized, brains were rapidly removed, and coronal sections (2‐mm thickness) were prepared using a mouse brain slicer (Muromachi). Total RNA was extracted from brain punches (hippocampal CA1 region) using a NucleoSpin RNA XS Kit (Macherey‐Nagel) according to the manufacturer's instructions. Subsequent steps were performed in the same method as in Section 2.7. The brains of fasted mice were stained by the frozen section method.^44^, ^51^ For AC3/MCHR1 staining in primary cilia, 8‐µm sections were heated at 70°C for 20 min in Histo VT One for antigen retrieval and blocked with Blocking One Histo (Nacalai Tesque) for 5 min. After three washes with PBS, the sections were incubated with rabbit anti‐human AC3 (C‐20; sc‐588; Santa Cruz Biotechnology; 1:500) and goat anti‐human MCHR1 (1:300) primary antibodies for 16–24 h at 4°C. The bound antibodies were detected by incubation with Alexa Fluor 488‐conjugated donkey anti‐rabbit IgG or Alexa Fluor 546‐conjugated donkey anti‐goat IgG secondary antibodies for 1 h at 25°C. The primary and secondary antibodies were diluted in a solution of 5% Blocking One Histo and 0.5% Triton X‐100 in PBS. The sections were counterstained with DAPI for 10 min and mounted with VECTOR Shield. Images were obtained with a BZ‐9000 fluorescence microscope with a 40× objective.

### 2.15 Statistical analysis

Statistical tests were performed in the StatView software (SAS Institute Inc.). Student's *t*‐test was used for comparison between two groups, and one‐way analysis of variance (ANOVA) was used for multi‐group comparison. Moreover, the data including the two factors were analyzed by two‐way ANOVA. Post hoc test (Tukey–Kramer method) was performed as needed based on the results of ANOVAs. A *p*‐value of 0.05 was set as the level of significance for all analyses (*p < 0.05, **p < 0.01, ***p < 0.001).

## 3 RESULTS

### 3.1 Validation of signaling pathways for MCHR1‐mediated cilia length shortening

Clonal cells expressing MCHR1:EGFP were grown to ~90% confluency in serum containing medium and subjected to serum starvation for 24 h to induce ciliogenesis. As previously reported, treatment of ciliated cells with 1 µM MCH for 6 h resulted in a distinct reduction in cilia length by approximately 32% (Table 1).^45^, ^47^ Pharmacological characterization of the phenomenon indicates that the MCHR1‐Gi/o‐Akt signaling pathway is the main contributor to the initial stage of MCH‐induced cilia length reduction in MCHR1:EGFP.^45^, ^47^ In fact, compounds including the protein kinase A inhibitors (H‐89, KT5720, Rp‐cAMP), Epac inhibitors (ESI‐09, CE3F4), MEK1/MEK2 inhibitor U0126, autophagy inhibitors (leupeptin, chloroquine diphosphate), and the proteasome inhibitor MG132 did not affect the MCHR1‐mediated cilia length reduction.^45^ However, the blocking effect of the Akt inhibitor on cilia shortening was not as potent as that of PTX treatment. This suggests that there are more signaling elements downstream of Gi/o involved in the initial step of MCHR1‐mediated cilia shortening. Therefore, we aimed to identify other putative signals using pharmacological interventions that we have not tested previously.

**TABLE 1:** Effects of different pharmacological treatments on MCHR1‐induced ciliary shortening

Drug (target) (Reference)	Concentration (µM)	Pretreatment (h)	Drug −MCH (µm)	Drug 1 µM MCH (µm)	+MCH/−MCH ratio (%)
No treatment			4.76 ± 0.07	3.25 ± 0.03^a^	68.3 ± 1.1
AG1478 (EGFR)^53^	10	0.5	4.78 ± 0.08	3.15 ± 0.07^a^	66.0 ± 1.2
Y27632 (Rho)^54^	10	1	4.47 ± 0.15	3.07 ± 0.06^a^	68.7 ± 1.8
ZCL278 (cdc42)^55^	50	1	4.04 ± 0.05	2.83 ± 0.05^a^	66.3 ± 2.0
EHT1864 (Rac‐1)^56^	10	1	4.69 ± 0.05	3.97 ± 0.02^a^	84. 6 ± 0.5^b^
NSC23766 (Rac‐1)^57^	3	1	4.72 ± 0.04	3.94 ± 0.04^a^	83.3 ± 0.62^b^
SB203580 (p38)^58^	30	0.5	4.18 ± 0.06	2.68 ± 0.08^a^	64.1 ± 0.90
SP600125 (JNK)^59^	3	0.5	4.57 ± 0.10	3.77 ± 0.15^a^	82.5 ± 1.95^b^
Akti1/2 (Akt)^45^, ^47^	3	0.5	4.73 ± 0.11	4.08 ± 0.20^a^	86.3 ± 2.4^b^

Note

hRPE1 clone cells expressing MCHR1:EGFP were pretreated with individual pharmacological drugs at various times and concentrations as indicated. The cells were then incubated with the drug alone or MCH plus drug for 1 h. After agent removal by washing with serum‐free medium, the cells were incubated without an agent for 5 h, fixed, and immunostained with an anti‐GFP antibody. The changes in the MCHR1‐positive ciliary length were determined using PhotoRuler Ver. 1.1 software under a fluorescence microscope. All experiments were independently performed at least three times. The data represent means ± SEM of three independent experiments (>100 cells per experiment).

^a^ *p* < 0.01, significant difference relative to no addition control by Student's *t*‐test.

^b^ *p* < 0.001, significant difference from +/− ratio in no treatment control from by Student's *t*‐test.

**FIGURE 1:**
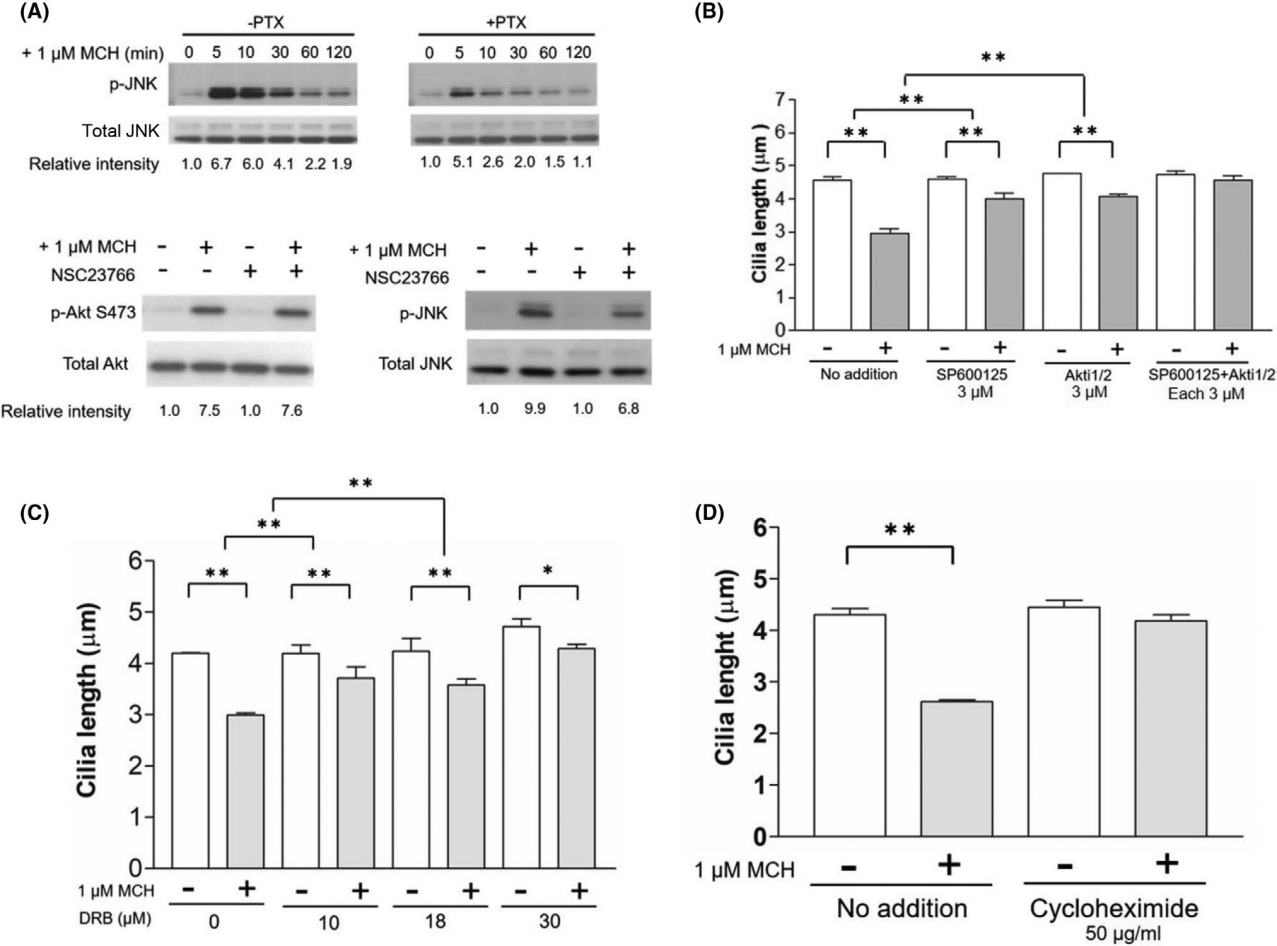
Detailed analysis reveals a novel signaling pathway that led to MCH‐caused cilia shortening in MCHR1:EGFP‐expressed clone cells. (A) Effects of MCH on JNK activation. Western blot analysis shows that MCH‐stimulated JNK phosphorylation in a manner that is partially sensitive to PTX (upper panels). Rac‐1 inhibitor, NSC23766, does not affect the level of Akt phosphorylation, but partially suppresses that of JNK phosphorylation (bottom panels). (B) Effect of Akti1/2 or/and SP600125 treatment on MCHR1‐mediated cilia shortening. The cells are treated with phosphorylated JNK inhibitor (SP600125) and/or Akt inhibitor (Akti1/2) as described in Table 1, and the effect on MCH‐caused cilia shortening is evaluated. Both Akti1/2 and SP600125 suppressed the MCH‐mediated cilia shortening. The co‐treatment with both drugs has almost completely blocked the MCHR1‐mediated cilia shortening. Significance is evaluated using the two‐way ANOVA and Tukey–Kramer method (**p < 0.01). (C) Effect of transcription and translation‐related pharmacological drugs on MCHR1‐mediated cilia shortening. The cells are treated with MCH in the presence of the transcription inhibitor, 5,6 dichloro‐β‐D‐ribofuranosyl benzimidazole (DRB). MCH‐induced cilia length reduction is significantly suppressed in the presence of DRB (left panel). Furthermore, the cells are treated with MCH in the presence of protein synthesis inhibitor, cycloheximide (CHX). The extent of cilia shortening with CHX is strongly blocked compared with the absence of CHX (right panel). The significance of the test is evaluated using the two‐way ANOVA and Tukey–Kramer method (*p < 0.05, **p < 0.01)

The activation of GPCRs can lead to interactions with the transactivating epidermal growth factor receptor (EGFR).^52^ This could explain some of the non‐Akt‐related ciliary shortenings observed when MCHR1 is expressed. Treatment with the EGFR inhibitor AG1478,^53^ however, did not influence MCH‐induced ciliary shortening, suggesting that the effect of MCH on cilia shortening is not caused by off‐target EGFR activation (Table 1). Next, we examined the effects of the RhoGTPase pathway using Rho‐kinase inhibitor Y27632, Cdc42 inhibitor ZCL278, and Rac‐1 inhibitors EHT1864 and NSC23766^54^, ^55^, ^56^, ^57^ (Table 1). Inhibition of Rac‐1 by the two inhibitors significantly blocked the MCH‐induced shortening of cilia (EHT1864: *p* < 0.001, NSC23766: *p* < 0.001). We then examined the MAPK family, since we have reported that ERK activation is not related to MCHR1‐mediated cilia shortening as described above. As expected, MCH‐induced cilia shortening was also unaffected by the p38 MAPK inhibitor SB203580.^58^ Nonetheless, it was significantly affected by the inhibition of c‐Jun N‐terminal kinase (JNK) MAPK inhibitor SP600125^59^ (*p* < 0.001). Western blot analysis confirmed the MCH‐stimulated JNK phosphorylation in MCHR1:EGFP clone cells in a manner that was partially sensitive to PTX (Figure 1A, upper panel). Furthermore, MCH‐induced JNK activation, but not Akt activation, was partially blocked by the Rac‐1 inhibitor (Figure 1A, below). This suggests that the Rac‐1‐JNK pathway is involved in the initial stage of ciliary shortening in MCHR1:EGFP. Finally, simultaneous treatment with Akt and JNK inhibitors almost completely blocked MCHR1‐mediated cilia shortening (no addition: *p* = 0.0064, SP600125+Akti1/2: n.s.) (Figure 1B). Collectively, our data indicated that among diverse intracellular signaling pathways, the initiation of MCH‐induced cilia shortening depends on two parallel signaling events: Gi/o‐dependent Akt activation and Gi/o‐dependent JNK activation mediated by Rac‐1.

Next, to examine the mode of MCHR1‐mediated cilia shortening, MCHR1:EGFP clone cells were treated with MCH in the presence or absence of a transcription inhibitor, 5,6 dichloro‐β‐D‐ribofuranosyl benzimidazole (DRB). MCH‐induced reduction in cilia length was significantly suppressed in the presence of DRB (control vs. DRB 10 µM: *p* = 0.0011) (Figure 1C, left). To further investigate whether cilia shortening requires post‐transcriptional processing, the cells were treated with MCH in the presence or absence of a protein synthesis inhibitor, cycloheximide (CHX). In the presence of CHX, the extent of cilia shortening was strongly blocked compared to that in the absence of cycloheximide (Figure 1C, right). The percentage of cilia shortening caused by MCH for 6 h was 60.1 ± 3.2% of basal (medium alone). Meanwhile, that caused by MCH combined with CHX was 94.3 ± 6.3% of CHX alone. Overall, our data suggest that MCH‐induced cilia reduction requires upregulated protein synthesis, likely mediated by transcriptional events.

### 3.2 RNA‐seq analysis reveals candidate genes regulated through MCH‐ciliary MCHR1 activation

To identify mRNAs that are regulated during MCH‐induced cilia shortening, we conducted a whole‐transcriptome analysis of total RNA sequencing (RNA‐seq) from MCHR1:EGFP clone cells subjected to 1 µM MCH treatment for 2 h, as this time point reliably displayed ciliary shortening in this cell model. The cells treated with MCH showed an upregulation of 424 genes and a downregulation of 112 genes compared with static control cells (adjusted twofold change). The full list is shown in Table S5. In addition to cilia length‐associated Akt and JNK phosphorylation (Figure 1B), MCH elicited diverse pathways, including receptor internalization, Ca^2+^ mobilization, ERK phosphorylation, and inhibition of cyclic AMP accumulation in ciliary MCHR1‐expressing hRPE1 cells, in‐line with the reports of previous studies on MCHR1 activation signaling.^45^ To determine the candidate genes associated with cilia length shortening, we cross‐referenced our list with other systematic studies. These included cilia databases,^60^, ^61^, ^62^ cilia‐based proteomic analyses,^63^, ^64^, ^65^, ^66^, ^67^ and functional genomic screens involved in ciliogenesis control^32^ (Table 2). We identified 10 genes (STK38L, TUBB2A/B, CCNO, ARL13B, RFX2, RAB23, TUBA1C, CEP170B, and SPATA7) that are established or candidate ciliopathy genes. Among them, CCNO is thought to be specifically required for generation of multiciliated cells via centriole amplification.^68^ Thus, CCNO may also be an important regulator in monociliated cells such as hRPE1 cells. Two other genes, RGS3 and PPKAG2, modulate cilia length in hRPE1 cells,^32^ with RGS3 being a GTPase that attenuates and modulates the signaling of GPCRs. Similarly, we found that other RGS proteins, RGS2 and RGS4, were highly upregulated following MCH treatment. The transcription factor ATF3, which was also highly upregulated in our list, modulated ciliogenesis in mIMCD3 cells.^32^ ATF3 forms the activator protein 1 complex by interacting with other transcription factors, including MAFF and FOSB, and thus regulating the expression of downstream genes. Among the upregulated genes, both MAFF and FOSB were highly upregulated by 17‐ and 44‐fold, respectively. Of the upregulated genes, the gene products of PTGS2, CNN3, AKAP12, HSPA5, ZYX, ATP1B1, TAGLN, EZR, RAI14, and PDLIM5 were validated as ciliary proteins by proteomic analyses.^63^, ^64^, ^65^, ^66^, ^67^ Of these 10 genes, PDLIM5 has been identified as a novel ciliary component in two previous systematic studies related to cilia.^66^, ^67^ Out of the remaining transcripts, we identified three genes (ARC, RAB3B, and BMF) that may control vesicle trafficking or are active regulators of the cytoskeleton. This is because changes in ciliary length require regulation of the cytoskeleton, IFT, and cargo loading.^10^, ^14^

**TABLE 2:** Search for genes involved in MCH‐MCHR1‐mediated cilia shortening by RNA‐seq analysis

Gene names	No MCH (FPKM)	MCH 2 h (FPKM)	MCH 2 h/No MCH fold change	Cross‐reference	Protein function
FOSB	0.86	81.8	44.75		Transcription factor
RGS2	8.09	211.07	22.55		GPCR signaling
MAFF	3.66	80.82	17.54		Transcription factor
PTGS2	0.61	26.32	16.96	Significantly enriched ciliary protein^66^	Prostaglandin‐endoperoxide synthase 2
ATF3	0.85	28.63	15.94	Knockdown inhibits ciliogenesis^65^	Transcription factor
RGS4	52.15	457.03	8.59		GPCR signaling
ARC	0.01	7.26	8.11		Activity‐regulated cytoskeleton‐associated protein
STK38L	7.62	41.95	4.89	Candidate ciliopathy gene	Protein serine/threonine kinase
TUBB2A	8.58	42.58	4.45	Candidate ciliopathy gene	Beta‐tubulin subtype
RGS3	64.3	232.5	3.48	Knockdown facilitaes cilium extension^32^	GPCR signaling
TUBB2B	3.91	16.3	3.48	Candidate ciliopathy gene	Beta‐tubulin subtype
CNN3	95.06	318.05	3.34	Significantly enriched ciliary protein^63^, ^66^	Actin, calmodulin and tropomyosin associated protein
AKAP12	8.56	30.55	3.19	Significantly enriched ciliary protein^66^	A‐kinase anchoring protein
HSPA5	267.25	818.33	3.13	Significantly enriched ciliary protein^66^	Heat shock protein
ZYX	85.13	248.54	2.86	Significantly enriched ciliary protein^66^	Focal adhesion protein
ATP1B1	19.56	59.66	2.8	Significantly enriched ciliary protein^66^	Sodium pump subunit
CCNO	6.77	19.67	2.59	Established ciliopathy gene (primary ciliary dyskinesia)	Regulation of the cell cycle
PRKAG2	6.79	19.33	2.54	Knockdown facilitaes cilium extension^65^	5′‐AMP‐activated protein kinase subunit
TAGLN	203.42	524.63	2.54	Significantly enriched ciliary protein^64^, ^66^	Actin‐binding protein
EZR	55.2	145.08	2.51	Significantly enriched ciliary protein^64^, ^66^	Linker between the plasma membrane and the cytoskeleton
ARL13B	2.25	6.43	2.32	Established ciliopathy gene (Joubert syndrome)	Ras superfamily of GTPase
RAI14	13.67	33.11	2.27	Significantly enriched ciliary protein^66^	Actin‐binding protein (testis)
RFX2	1.11	3.64	2.27	Candidate ciliopathy gene	Transcription factor
PDLIM5	49.33	113.5	2.25	Significantly enriched ciliary protein^66^, ^67^	Scaffold protein
RAB23	2.2	5.95	2.19	Established ciliopathy gene (Carpenter syndrome)	Rab GTPase
RAB3B	1.79	4.94	2.16		Rab GTPase
TUBA1C	87.77	191.45	2.12	Candidate ciliopathy gene	Alpha‐tubulin subtype
CEP170B	4.84	11.11	2.04	Candidate ciliopathy gene	Centrosomal protein
SPATA7	10.53	3.63	−2.49	Established ciliopathy gene (Leber congenital amaurosis and juvenile retinitis pigmentosa)	Maintenance of both rod and cone photoreceptor cells
BMF	49.73	2.52	−14.36		Proapoptotic protein, Sequestered to myosin V motors

Note

Differences in gene expression were investigated between untreated and treated MCHR1:EGFP clonal cells with 1 µM MCH for 2 h. Genes related to primary cilia and cytoskeleton were selected from genes that fluctuated more than twice in the normalized value (FPKM). For example, MCH 2 h/No MCH.fc has “2” means that probe is 2 fold up‐regulated in test sample.

If MCH 2 h/No MCH.fc has “−2”, probe is 2 fold down‐regulated in MCH 2 h sample.

**FIGURE 2:**
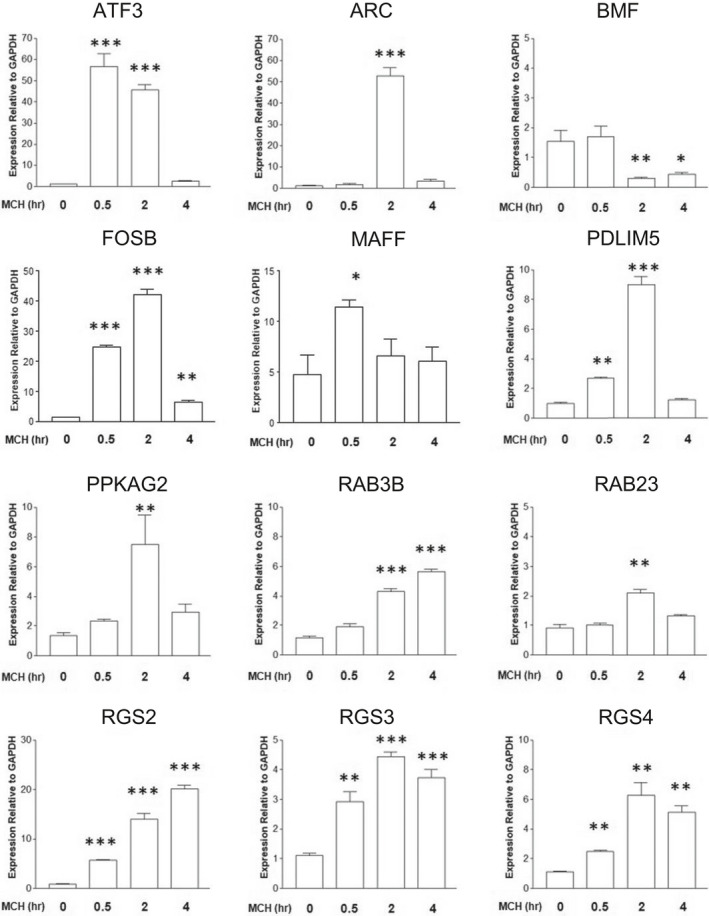
qRT‐PCR analysis reveals the time course of expression levels for the candidate genes involved in MCHR1‐mediated cilia shortening. Relative gene expression levels of MCHR1:EGFP clone cells treated with 1 µM of MCH at 0.5, 2, and 4 h or untreated as controls (0). The numbers of target genes are normalized by that of a housekeeping gene (GAPDH) in each sample (*n* = 4). Significant differences relative to control are determined using the Tukey–Kramer method (*p < 0.05, **p < 0.01, ***p < 0.001)

Among these 30 genes, we focused on 12 genes as strong candidates to participate in MCHR1‐mediated cilia length regulation: transcription factors (FOSB, ATF3, and MAFF), RGS proteins (RGS2, RGS3, and RGS4), the significantly enriched ciliary protein (PDLIM5) reported by two independent laboratories,^66^, ^67^ trafficking‐related proteins (RAB3B, RAB23, ARC, and BMF), and a possible modulator of ciliary dynamics (PRKAG2). To corroborate these candidates identified through RNA‐seq analysis, the time course of expression levels (0.5, 2, and 4 h) was determined by qRT‐PCR analysis of each gene in three independent experiments (Figure 2 and Table S6). Changes in gene expression following MCH treatment for 2 h were validated in 11 genes. Meanwhile, MAFF showed a reduction in change amplitude in qRT‐PCR compared with that in RNA‐seq analysis. The time course profile revealed that ATF3 and FOSB were highly upregulated at 0.5 and 2 h. On the other hand, six genes (RAB23, RGS3, RGS4, PPKAG2, PDLIM5, and ARC) showed maximal expression after MCH treatment for 2 h.

**FIGURE 3:**
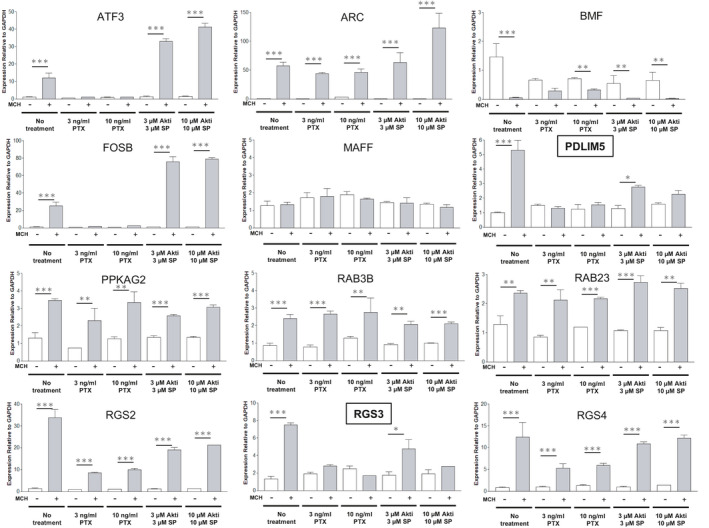
RGS3 and PDLIM5 are positioned as promising candidates for the MCH‐MCHR1‐induced cilia shortening. Effect of PTX, Akti1/2 (Akti), and SP600125 (SP) treatment on each gene fluctuated after MCH treatment. The cells are pretreated with or without the drug and then incubated with or without 1 µM MCH for 2 h. The amounts of target genes are normalized by that of a housekeeping gene (GAPDH) in each sample (*n* = 4). Significance tests compared to controls (no treatment) in each treatment group are performed using the two‐way ANOVA and Tukey–Kramer method (*p < 0.05, **p < 0.01, ***p < 0.001). Only RGS3 and PDLIM5 (framed bold). Have its mRNA upregulation limited by treatment to PTX and Akti/SP

Using qRT‐PCR analysis, we aimed to identify the genes that are directly involved in shortening cilia length, as opposed to other constitutive ciliary and non‐ciliary functions. To achieve this, we leveraged the above results showing that both Gi/o and Akt/JNK activation is necessary for the ciliary shortening effect of MCH (Figure 1B). Our rationale is that any gene critical for MCH‐induced ciliary shortening should have its upregulation limited by exposure to a Gi/o inhibitor (PTX) and Akt/JNK inhibitors (Akti1/2 and SP600125). Of the genes whose expression was upregulated after 2 h of exposure to MCH, only the RGS3 and PDLIM5 genes met the criteria of being significantly blocked at both steps (Figure 3 and Table S7), and thus positioning those genes as promising candidates to mediate cilia length shortening by interacting with MCH‐MCHR1.

### 3.3 PDLIM5 is an Akt/JNK‐related modulator of MCHR1‐mediated cilia shortening

**FIGURE 4:**
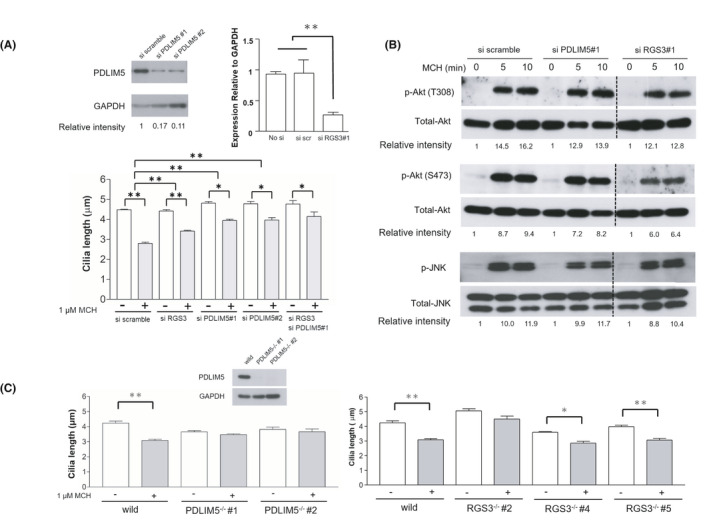
Reduced PDLIM5 products impair cilia shortening mediated by MCHR1. (A, B) Knockdown experiment using siRNA transfection. (A) Transfection of MCHR1:EGFP clone cells with two independent siRNAs of PDLIM5 achieves 90% reduction of protein products (upper left panel). Although the protein level of RGS3 cannot be measured because of the lack of a commercially available anti‐RGS3 antibody, it is confirmed by qRT‐PCR that the mRNA expression level is reduced by approximately 70%. The significance test is evaluated using the Tukey–Kramer method (upper right panel). Cells with reduced levels of PDLIM5 and/or RGS3 are used to evaluate cilia shortening due to MCH. Interference of RGS3 and PDLIM5 functions, both reduce the magnitude of ciliary shortening after MCH exposure. There is no additive effect between RGS3 and PDLIM5 interference. Significance is evaluated using the two‐way ANOVA and Tukey–Kramer methods (*p < 0.05, **p < 0.01) (bottom panel). (B) Signaling status in cells transfected with RGS3 siRNA and PDLIM5 siRNA by western blotting. Knockdown of RGS3 and PDLIM5 does not affect the MCH‐induced Akt (T308 and S473) and JNK phosphorylation. (C) Analysis using knockout cells produced using the CRISPR/Cas9 method. PDLIM5 and RGS3 knockout cells (PDLIM5^−/−^, RGS3^−/−^) transiently transfected with MCHR1:EGFP plasmid show significantly reduced MCHR1‐mediated cilia shortening. In particular, the constitutive deletion of PDLIM5 results in the drastic prevention of MCHR1‐mediated cilia shortening after MCH treatment. In PDLIM5 knockout cells, the western blotting analysis shows the absence of PDLIM5 protein. Significance relative to MCH is determined using the two‐way ANOVA and Tukey–Kramer method (*p < 0.05, **p < 0.01)

Next, we further characterized whether RGS3 and PDLIM5 are required for MCHR1‐mediated cilia shortening by employing siRNA to knockdown genes. Transfection of MCHR1:EGFP cells with two independent siRNAs for PDLIM5 successfully reduced the levels of protein products (Figure 4A, upper left panel) and resulted in significantly blocked MCH‐induced cilia shortening (si scramble vs. si PDLIM5#1: *p* = 0.0044, si scramble vs. si PDLIM5#2: *p* = 0.0081), while control siRNAs had no effect (Figure 4A, bottom panel). These responses in the siRNA‐treated cells were complementary under the exogenous PDLIM5 gene expression (Figure S1), suggesting that the PDLIM5 genes are necessary for the cilia length shortening caused by MCH. The results for RGS3 were less clear, as the lack of commercially available anti‐RGS3 antibodies precluded the determination of the protein levels of RGS3. Even though, a substantial decrease was observed in RGS3 gene expression following the use of siRNAs (si scramble vs. si RGS3#1: *p* = 0.0024) (Figure 4A, upper right panel). The interference of RGS3 function decreased the magnitude of ciliary shortening after MCH exposure (si scramble vs. si RGS3: *p* = 0.0031) (Figure 4A, upper right panel). However, this effect is significantly smaller than that of PDLIM5, and there is no additive effect between RGS3 and PDLIM5 interference. This suggests a degree of redundancy between these two intermediaries.

Our data suggest that both RGS3 and PDLIM5 are upregulated by MCH in a Gi/o‐Akt‐ and Gi/o‐JNK‐dependent manner. To investigate whether the reduced RGS3 and PDLIM5 products in MCHR1:EGFP clone cells were attributable to Akt and JNK activation, we analyzed the signaling status in the cells transfected with RGS3 siRNA and PDLIM5 siRNA by western blotting. As expected, knockdown of RGS3 and PDLIM5 did not inhibit MCH‐induced Akt (T308 and S473) and JNK phosphorylation (Figure 4B). This suggests that the signaling pathway at the initial stage of cilia length shortening (MCHR1‐Akt and MCHR1‐JNK) is retained in cells even if the levels of RGS3 mRNA or PDLIM5 product decreased.

To further confirm whether RGS3 and PDLIM5 are indeed required for MCHR1‐induced cilia reduction, we disrupted the RGS3 and PDLIM5 genes in wild hRPE1 cells with a uniform genetic background using the nonhomologous end––joining––mediated targeting method called ObLiGaRe.^48^ In this method, co‐transfection of CRISPR/Cas9 and drug‐resistant gene cassette vectors tagged with the CRISPR/Cas9 site located in the genome into hRPE1 cells enabled the generation of RGS3––and PDLIM5––knockout cell clones. Western blotting analysis demonstrated the absence of PDLIM5 products in the two different clones of PDLIM5^−/−^ hRPE1 cells (Figure 4C, insertion). These clones were then transiently transfected with MCHR1:EGFP plasmid, grown for 24 h in a serum‐free medium to allow primary cilium formation, and the capacity for MCH‐induced cilia shortening was evaluated. The constitutive deletion of PDLIM5 resulted in a drastic reduction in MCHR1‐mediated shortening after 6 h of MCH treatment (Figure 4C, left panel). The lack of response to MCH in the PDLIM5^−/−^ hRPE1 cells were rescued under the exogenous PDLIM5 gene expression (Figure S2), confirming the importance of PDLIM5 for the cilia length shortening caused by MCH.

A procedure similar to that described above was performed to generate clones of RGS3^−/−^ cells. The effectiveness of the knockout model could not be verified because of the lack of suitable antibodies. Of the three generated clones, only one clone (clone #2) displayed a significant decrease in MCH‐induced ciliary shortening, while the other two clones (clones #4 and #5) showed a larger nucleus compared to that in wild type cells (Figure 4C, right panel). Due to the technical difficulties in examining the RGS3 function, further experiments in that gene were discontinued to focus on PDLIM5, given the strong support from our data for mediating MCHR1‐linked ciliary shortening.

To ensure that PDLIM5 deletion did not compromise constitutive cilia formation in hRPE1 cells, the number and length of primary cilia were investigated in PDLIM5^−/−^ serum, starved for 24 h (assembling cilia), and refed with serum for 24 h (disassembling cilia). Quantification of ciliation frequencies showed no differences between PDLIM5^−/−^ hRPE1 clone #2 and wild hRPE1 cells (Figure S3). These results suggest that hRPE1 cells without PDLIM5 can conserve the basic signaling cascades of ciliogenesis following serum addition and starvation, even though PDLIM5 has been identified as a component of the ciliary compartment. PDLIM5, which consists of a PDZ domain and three LIM domains, is a member of the PDZ‐LIM family of acting scaffolds. PDLIM5 binds to a variety of partners through its PDZ and LIM domains and plays an important role in multiple tissues and cell types, including neuronal cells. However, the functional significance of PDLIM5 in ciliary dynamics is still lacking. Therefore, we characterized the role of PDLIM5 and its relationship with MCH.

**FIGURE 5:**
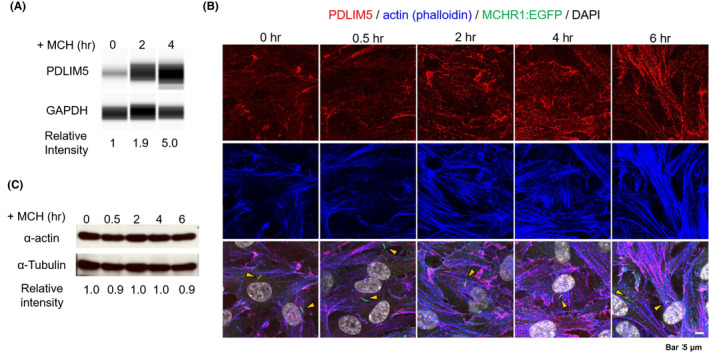
Dynamics of PDLIM5 protein and actin polymerization are associated with MCH stimulation. (A) The expression level of PDLIM5 protein using WES, which is a capillary‐based western blot analysis. PDLIM5 protein increases in an MCH time‐dependent manner. (B) Representative images of immunofluorescence staining for PDLIM5 (red), MCHR1:EGFP (green), and actin (blue) in MCHR1:EGFP cells cultured with 1 µM MCH. Nuclei are labeled with DAPI (white). When the cells are treated with 1 µM MCH, the co‐upregulation of PDLIM5 and actin polymerization is observed depending on the MCH stimulation time. Cilia (arrows) are labeled with EGFP antibodies. (C) Analysis of the amount of alpha‐actin by western blot analysis. The amount does not increase when the cells are treated with MCH up to 6 h

First, since MCH treatment caused a transient increase in PDLIM5 transcripts (Figure 2), we examined whether this upregulation translated into higher protein synthesis. MCH treatment for 2 h increased the relative intensity of PDLIM5 to 1.9‐fold of basal (medium alone) and 5.0‐fold after 4 h (Figure 5A), corroborating the effective upregulation of PDLIM5 by MCH. Next, we examined the subcellular localization of PDLIM5 upon stimulation with MCH. PDLIM5 is associated with F‐actin structures^69^, ^70^ via the PDZ domain of PDLIM.^71^ Previously, we revealed that MCH treatment leads to actin polymerization in MCHR1:EGFP clone cells.^47^ Thus, we considered whether co‐localization of PDLIM5 with actin molecules would be altered after MCH treatment. Without MCH, we found that some PDLIM5 co‐localized with F‐actin in the peripheral regions of the cell. We were unable to detect a discrete PDLIM5 localization within the MCHR1‐positive cilia or cilium bases, although PDLIM5 has previously been identified as a ciliary membrane protein.^66^, ^67^ When cells were treated with MCH, co‐upregulation of PDLIM5 with actin polymerization was observed without increased amounts of alpha‐actin (Figure 5B,C). Collectively, these results indicate that PDLIM5 is functionally involved in MCHR1‐induced shortening of cilia length mediated by actin polymerization.

**FIGURE 6:**
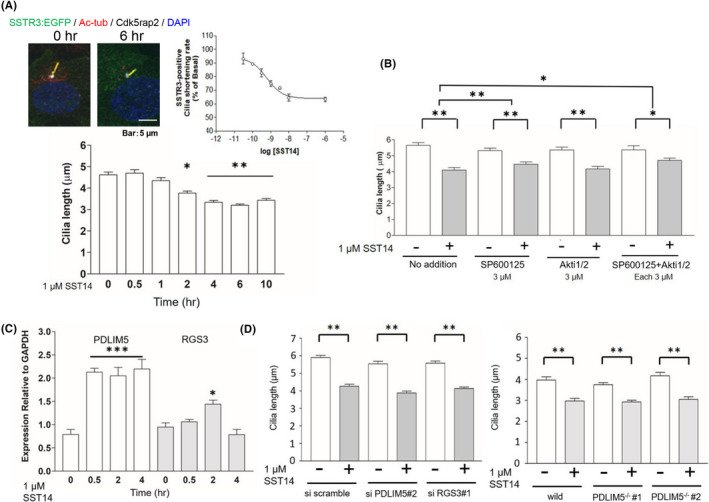
PDLIM5 is not a positive regulator for cilia shortening through SST14‐SSTR3 interaction. (A) Representative images of immunofluorescence staining for SSTR3:EGFP (green), Ac‐tub (red), and centrosome marker Cdk5rap2 (white) in SSTR3:EGFP clone cells with or without 1 µM SST14. Nuclei were labeled with DAPI (blue). SST14 shortens the primary cilia by approximately 25% (upper left panel) in a concentration‐dependent manner (EC50 = 0.61 nM) (upper right panel). SST14 shortens the primary cilia for 2 h with a maximum effect of 6 h (bottom panel). The significance test for 0 h is evaluated using the Tukey–Kramer test (*p < 0.05, **p < 0.01). (B) SSTR3:EGFP clone cells are pretreated with SP600125 and/or Akti1/2, and the effect on SST14‐caused cilia shortening is evaluated. In the cells treated with Akti1/2 and/or SP600125, the extent of SST14‐caused cilia shortening is significantly suppressed as well as MCH‐caused cilia shortening in MCHR1:EGFP. The significance test for SST14 is evaluated using the two‐way ANOVA and Tukey–Kramer test (*p < 0.05, **p < 0.01). (C) Quantification of PDLIM5 and RGS3 mRNA expression levels to SST14 treatment. The response pattern of each gene is different from that of MCH‐MCHR1 (Figure 2). PDLIM5 increased continuously from 0.5 h after SST14 addition, while only a slight increase in RGS3 is observed 2 h after SST14 addition. The significance test for 0 h is evaluated using the Tukey–Kramer method (*p < 0.05, ***p < 0.001). (D) Analysis of SSTR3‐mediated cilia shortening in cells showed less expression of PDLIM5 or RGS3 products. Knockdown of RGS3 or PDLIM5 mRNA by siRNA transfection does not affect the cilia shortening via SST14‐SSTR3 (left panel). In PDLIM5 knockout cells (PDLIM5^−/−^) transiently transfected with SSTR3:EGFP plasmid, SST14 treatment in SSTR3‐positive primary cilia is shortened, as in the control (wild) (right panel). The significance test for SST14 is evaluated using the two‐way ANOVA and Tukey–Kramer method (**p < 0.01)

Next, we determined whether PDLIM5 is a universal mediator of GPCR‐induced ciliary shortening or responds specifically to MCH‐MCHR1 binding. To test this, we chose somatostatin receptor 3 (SSTR3) and somatostatin 14 (SST14), which also induces a significant reduction in cilia length in cells transiently transfected with SSTR3:EGFP.^45^ As expected, treatment of SSTR3:EGFP clone cells with 1 µM SST14 for 6 h resulted in a 31% reduction in cilia length (mean ± SEM: 4.62 ± 0.18 µm vs. 3.20 ± 0.10 µm) with an EC_50_ value of 0.61 nM (Figure 6A). Furthermore, SSTR3‐mediated cilia shortening was mediated via Akt‐ and JNK‐dependent signaling pathways (Figure 6B), as indicated by our pharmacological study. Despite these similarities, qRT‐PCR revealed that the time course profile of PDLIM5 expression was different between ciliary SSTR3‐mediated cilia shortening and MCHR1‐mediated cilia shortening (Figures 2 and 6C). Both PDLIM5 knockdown and knockout did not affect the ciliary SSTR3‐mediated cilia shortening (Figure 6D). Overall, these data suggest that PDLIM5 is a positive regulator of cilia shortening through MCHR1‐ but not via SSTR3‐mediated pathways. In addition, the level of RGS3 expression by SST14 for 2 h was only 1.5‐fold (Figure 6C), and the SSTR3:EGFP clone cells transfected with RGS3 siRNA did not influence SST14‐induced shortening of cilia (Figure 6D, left panel). Therefore, the requirement of RGS3 in SSTR3‐mediated cilia shortening is less likely.

### 3.4 Alpha‐actinin 1/4 is a downstream target of PDLIM5 in MCHR1‐mediated cilia shortening

**FIGURE 7:**
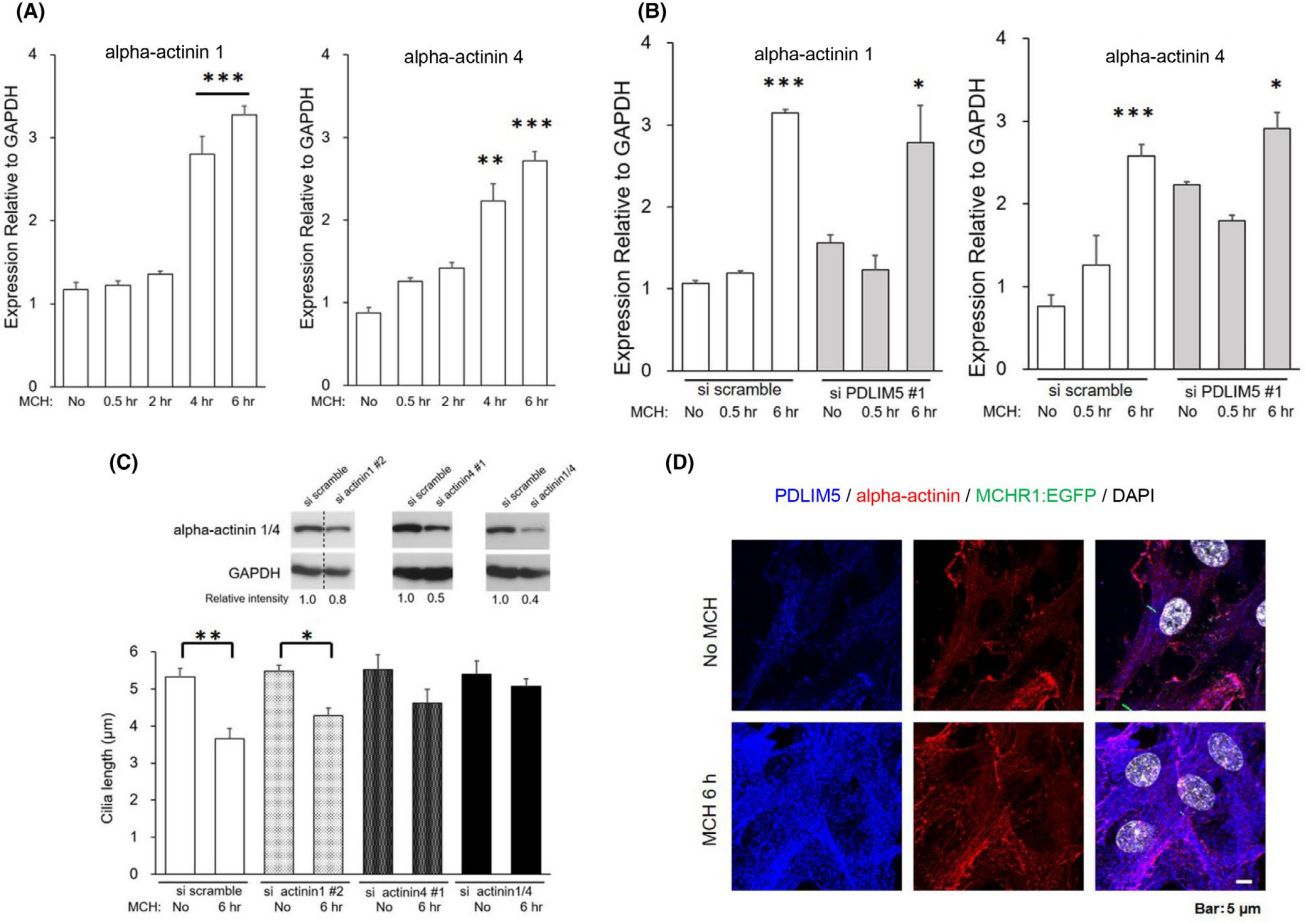
Alpha‐actinin 1/4 is the downstream target of PDLIM5 for MCHR1‐mediated cilia shortening. (A) Quantification of mRNA expression levels of alpha‐actinin 1/4 with MCH treatment in MCHR1:EGFP clone cells. The amounts of the target genes are normalized by that of a housekeeping gene (GAPDH) in each sample (*n* = 4). The amount of alpha‐actinin 1/4 mRNA significantly increases 4 h after MCH treatment. The significance test for no MCH is evaluated using the Tukey–Kramer method (**p < 0.01, ***p < 0.001). (B) Knockdown experiment using PDLIM5 siRNA transfection. In the control scramble, the alpha‐actinin 1/4 mRNA significantly increases after 6 h of MCH treatment (***p < 0.001), while this upregulation is attenuated by PDLIM5 knockdown (*p < 0.05). In each group, the significance test for no MCH is evaluated using the two‐way ANOVA and Tukey–Kramer method (*p < 0.05, ***p < 0.001). (C) Knockdown experiment using alpha‐actinin 1/4 siRNA transfection. Co‐knockdown with alpha‐actinin 1 and 4, which leads to a ~60% reduction of protein (insertion). Knockdown of alpha‐actinin 1 or 4 attenuates MCH‐induced cilia shortening. In addition, alpha‐actinin 1 and 4 co‐knockdown markedly prevents cilia shortening. The significance test for no MCH is evaluated using the two‐way ANOVA and Tukey–Kramer method (*p < 0.05, **p < 0.01). (D) Subcellular localization of alpha‐actinin 1/4 in MCHR1:EGFP. Representative images of immunofluorescence staining for PDLIM5 (blue), alpha‐actinin (red), MCHR1:EGFP (green), and DAPI (white) in MCHR1:EGFP cultured with 1 µM MCH. When cells are treated with MCH for 6 h, co‐upregulation of alpha‐actinin 1/4 with PDLIM5 is detected both in filamentous structures and peripheral regions

As described above, PDZ‐LIM family members function as protein–protein modules that facilitate various cellular signaling mechanisms. PDZ‐LIM proteins, including PDLIM5, interact with alpha‐actinin 1/4,^69^, ^70^, ^71^ which is an F‐actin regulator by cross‐linking F‐actin and anchoring F‐actin to cell–cell and cell–matrix junctions.^72^ MCH‐induced upregulation of PDLIM5 is involved in actin polymerization (Figure 5B). Therefore, we assessed whether alpha‐actinin 1/4 functioning as an actin coordinator is the downstream target of PDLIM5 for MCHR1‐mediated cilia shortening. As shown in Figure 7A, treatment with MCH for 4 and 6 h increased the expression of alpha‐actinin 1 and 4 mRNA by more than twofold in MCHR1:EGFP clone cells. This effect was attenuated by PDLIM5 knockdown (changes in actinin 1 mRNA expression level after MCH 6 h stimulation, si scramble: *p* < 0.001, si PDLIM5: *p* = 0.0153; actinin 4: si scramble: *p* < 0.001, si PDLIM5: *p* = 0.0192) (Figure 7B). These results suggest a link between the alpha‐actinin 1/4 gene and PDLIM5. We then evaluated whether a functional link existed between ciliary shortening and alpha‐actinin 1/4. Both alpha‐actinin 1 knockdown and alpha‐actinin 4 knockdown significantly prevented MCH‐induced shortening of cilia. Co‐knockdown with alpha‐actinins 1 and 4, which led to a ~60% reduction in protein, profoundly prevented cilia shortening (si scramble: *p* = 0.0047, si actinin 1/4: n.s.) (Figure 7C). These results indicate the functional role of alpha‐actinin 1/4 in MCHR1‐mediated cilia regulation.

Next, we investigated the subcellular localization of alpha‐actinin 1/4 in cells. We found a distinct immunosignal of alpha‐actinin 1/4 in the peripheral regions of the cell and observed some co‐localization with PDLIM5. When cells were treated with MCH for 6 h, we detected a co‐upregulation of alpha‐actinin 1/4 with PDLIM5 in both filamentous structures and peripheral regions (Figure 7D). Although alpha‐actinin is localized at the base of the cilia,^66^ we could not confirm this in MCHR1‐positive ciliary bases with or without MCH. In addition, alpha‐actinin 1/4 knockdown in SSTR3‐expressing cells did not affect the SST14‐induced shortening of cilia (data not shown). Overall, these results suggest that appropriate signaling for MCH‐MCHR1‐induced cilia shortening relies on a PDLIM5 alpha‐actinin 1/4 combination, at least in part, achieved by actin polymerization.

To examine the possibility that PDLIM5 affects other downstream targets, we investigated the transcriptional coactivator yes‐associated protein (YAP), a novel PDLIM5‐binding protein.^71^ Another group reported the functional relationship between YAP activation and ciliary dynamics. That is where the YAP pathway is suggested to be a possible key component of transcriptional networks governing cilia growth control.^73^ Therefore, we investigated whether YAP is a significant candidate for PDLIM5‐binding proteins for cilia length control. However, our knockdown experiments using two independent siRNAs did not affect the cilia shortening mediated by MCHR1 (Figure S4). If YAP could be a member of the PDLIM5 scaffold protein complex in MCHR1‐expressing hRPE1 cells, this protein–protein interaction may not be involved in cilia length dynamics but other cellular functions.

**FIGURE 8:**
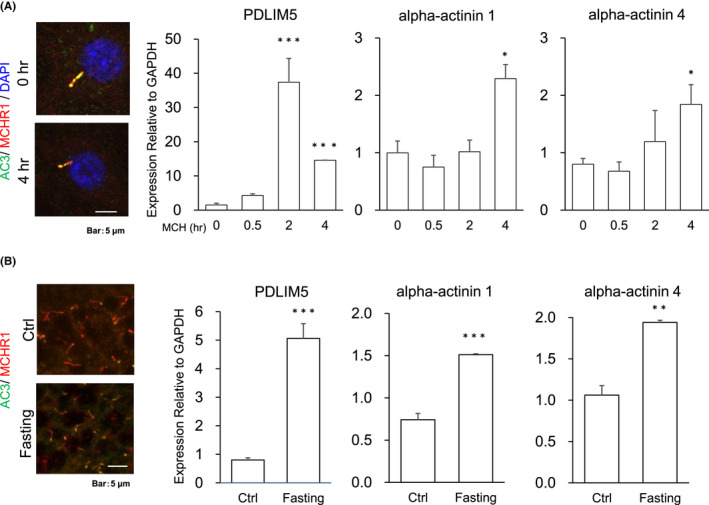
PDLIM5 and alpha‐actinin 1/4 are associated with MCH‐MCHR1 ciliary degeneracy in rat hippocampal primary neurons and mouse brain section. (A) Photomicrographs of cilia shortening after treatment of dissociated rat hippocampal neurons with MCH. The dissociated rat hippocampal neurons are treated with 10 nM MCH or untreated for 4 h. Primary cilia were co‐labeled with antibodies against AC3 (green) and MCHR1 (red). Nuclei are labeled with DAPI (blue). MCH treatment shortens the primary cilia by 25% (left pictures). Quantification of PDLIM5 and alpha‐actinin 1/4 mRNA expression levels of dissociated rat hippocampal neurons of 18 DIV. Neurons are treated with 10 nM MCH at three different time points (0.5, 2, and 4 h) or untreated as controls (0 h). The numbers of target genes are normalized by that of a housekeeping gene (GAPDH) in each sample (*n* = 4). Gene expression increases in 2 h for PDLIM5 and 4 h for alpha‐actinin 1/4. The significance test for 0 h is evaluated using the Tukey–Kramer method (*p < 0.05, ***p < 0.001) (right panels). (B) Photomicrographs of mouse hippocampal sections centered around the CA1 regions where primary cilia were co‐immunolabeled with antibodies against AC3 (green) and MCHR1 (red). Animals are either fed ad libitum (Ctrl, *n* = 4) or fasted for 48 h (Fasting, *n* = 4). Fasting, which increases the MCH expression in the lateral hypothalamus,^31^ shortens the primary cilia by approximately 10% (left pictures). Relative gene expression levels in the hippocampal CA1 region of fed and fasted mice evaluated by qRT‐PCR. The numbers of the target genes are normalized by that of a GAPDH in each sample (*n* = 3). Fasting results in an upregulation of PDLIM5 and alpha‐actinin 1/4 gene expression (Student *t*‐test, **p < 0.01, ***p < 0.001) (right panels)

Ciliary MCHR1 is endogenously and abundantly expressed in rodent hippocampal neurons.^43^, ^44^ Importantly, the application of MCH for 4 h in neurons caused MCHR1‐positive cilia shortening.^44^ Therefore, we examined whether endogenous MCHR1‐positive cilia shortening is associated with the upregulation of PDLIM5 expression in dissociated rat hippocampal neurons. PDLIM5 is localized in the dendrites of cultured hippocampal neurons.^74^, ^75^ However, in our immunohistochemical method, we could not detect PDLIM5‐specific signals in soma and neurite in primary cultures of 18 DIV using an anti‐PDLIM5 antibody with knockout‐mediated validation (Figure S5). We then validated the mRNA expression levels using qRT‐PCR. Treatment with MCH which caused a transient (2 h) and marked (−30‐fold) increase in PDLIM5 expression in dissociated rat hippocampal neurons at 18 DIV (0 h vs. MCH 2 h: *p* < 0.001, 0 h vs. MCH 4 h: *p* < 0.001) (Figure 8A). As regards alpha‐actinin 1 and 4 mRNA expression, MCH induced a moderately increased level after exposure for 4 h (actinin 1: *p* = 0.0134, actinin 4: *p* = 0.0246), but not after 2 h. Such MCH‐sensitive and time‐selective regulation of PDLIM5 and alpha‐actinin 1/4 is consistent with that observed in MCHR1‐expressing hRPE1 cells (Figures 2 and 7A). Lastly, we took advantage of some of our recent finding in vivo that MCHR1‐positive cilia length in the hippocampal CA1 region was significantly shortened in fasted mice compared with that in fed mice.^44^ When compared with the fed mice, mRNA expression of both PDLIM5 and alpha‐actinin 1/4 was upregulated in the CA1 region of the fasting mouse for 48 h (5‐fold and 1.5‐ to 2‐fold, respectively) (PDLIM5: *p* < 0.001, actinin 1: *p* < 0.001, and actinin 4: *p* = 0.0036) (Figure 8B). These results suggest a potential link between hippocampal MCHR1‐positive cilia shortening and upregulation of PDLIM5 and alpha‐actinin 1/4 in vivo as well as in vitro.

## 4 DISCUSSION

The study of primary cilia is an expanding field, propelled by the role played by these structures in coordinating signaling pathways, as ligands bind to ciliary receptors and efficiently relay signaling events, prompting the exchange of molecules between the cytoplasm and the isolated ciliary compartment.^2^, ^5^, ^6^, ^7^, ^8^, ^9^ Ciliary plasticity is an important element of this physiological signaling process, with cilia length being the most direct attribute modified by the binding of molecules to their receptors at the cilia surface.^10^ Identifying the molecular determinants of ciliary length is an essential first step in creating targeted interventions that may change the ciliary length without prompting off target and possibly unwanted effects of directly activating ciliary GPCRs.

MCH–MCHR1 binding at the ciliary membrane leads to consistently detectable ciliary shortening in both in vitro and ex vivo models.^44^ However, this is not the only effect of MCHR1 activation. This effect includes a myriad of downstream effects, as evident from the regulation of 536 genes in MCHR1‐expressing cells exposed to MCH for 2 h, as determined by RNA‐seq analysis in this study. Based on previous literature^45^, ^47^ and our discovery that Gi/o‐Akt and Gi/o‐JNK are necessary for the onset of MCHR1‐mediated cilia shortening, we were able to identify PDLIM5 as a suitable mediator of MCHR1‐induced ciliary shortening. PDLIM5 mRNA was transiently upregulated in a Gi/o‐, Akt‐, and JNK‐dependent manner in MCH‐treated cells, and its increased expression follows a temporal course that is consistent with that of MCHR1 ciliary shortening. Moreover, both its selective knockdown and knockout impair the ability of MCH to reduce the cilia length, without affecting the constitutive assembly and disassembly of primary cilia following the cell cycle.

PDLIM5 is a signal modulator that plays an important role in diverse cellular functions. According to recent studies, PDLIM5 may be involved in tumorigenesis, heart development, and mental illnesses.^76^, ^77^, ^78^, ^79^ In addition to acting as an actin‐binding agent, PDLIM5 binds to various signaling molecules. These include protein kinases, calcium channels, transcription factors, and other cytoskeletal proteins.^76^ Among them, we identified the actin‐bundling proteins alpha‐actinin 1 and 4 as key partners of PDLIM5 in MCHR1‐mediated cilia shortening. The knockdown of these proteins is also sufficient to block the MCH‐induced shortening of cilia. In our experiments, alpha‐actinin 1/4 mRNA expression was not upregulated at early time points. This suggests that the upregulated PDLIM5 initially binds to constitutive preexisting alpha‐actinin 1/4, and this interaction platform leads to the coordination of actin filaments that results in ciliary shortening. The idea that PDLIM5 and alpha‐actinin 1/4 contribute to actin mobilization following MCHR1 activation is supported by the results of our previous study, as we have demonstrated that actin polymerization (stabilized F‐actin) is required for cilia shortening via MCHR1.^47^ Since alpha‐actinin is a component of the actin cross‐linking functional module,^72^ this makes it a strong candidate in mediating the downstream effects of MCHR1 activation on cytoskeletal rearrangement. As PDLIM5, alpha‐actinin is a multitasking protein with roles ranging from forming tighter bridges between filaments, to acting as a versatile bridge between actin and cell adhesion and signaling proteins.^72^, ^80^, ^81^ Furthermore, a recent study has shown that alpha‐actinin is a critical postsynaptic docking protein for PSD‐95 and AMPA‐type glutamate receptor,^82^ and it plays a dual role by anchoring Ca^2+^ channel Ca_V_1.2 at the specific postsynaptic sites and at the same time boosting its open probability.^83^ Given the role of actin‐alpha‐actinin dynamics as an important cellular organizer, future characterization of other actin‐ and alpha‐actinin‐interacting proteins found in our RNA‐seq dataset might provide future targets for investigation. In fact, we found that our transcriptome results in Table S5 comprise at least 15 genes that are involved in actin‐related cellular processes. On the other hand, YAP knockdown was unsuccessful in preventing MCHR1‐mediated ciliary shortening, suggesting that there is some degree of selectivity in the downstream partners of PDLIM5 when activated through this route. More studies will be necessary to identify all suitable intermediaries in this cascade.

One of the surprising features observed in this study was the specificity of PDLIM5 and alpha‐actinin 1/4 with regard to MCHR1 activation. This was underscored by the continued SST14‐induced SSTR3‐mediated ciliary shortening when either PDLIM5 or alpha‐actinin 1/4 was knocked down using siRNA. The main reason for this observation is that the ciliary length control cascade is not a universal feature shared by all GPCRs that change ciliary morphology, but rather discrete cascades that allow each GPCR to contribute to the determination of the overall length of the cilium for one cell. The cilia length, therefore, is subject to the integration of multiple signals, rather than a binary state, irrespective of the signal received by the cilium. This increases the likelihood that pharmacological interventions on cilia length are possible without disrupting multiple signaling systems.

In addition to PDLIM5 and its partners, our qRT‐PCR analysis also showed that MCH significantly and transiently increased the mRNA levels of RGS3 in MCHR1‐expressing hRPE1 cells in a Gi/o and Akt/JNK‐dependent manner. The evidence obtained in this study suggests that RGS3 is also part of the MCHR1 cascade controlling ciliary length, including: (1) the RGS3 knockdown study suggested a plausible involvement of this protein in MCH‐induced cilia shortening; (2) RGS3 knockdown significantly blocked the MCH‐induced upregulation of alpha‐actinin 1/4 mRNA in MCHR1‐expressing cells (Figure S6); (3) addition of MCH in cultured rat hippocampal neurons 18 DIV caused a transient increase in RGS3 mRNA expression, which was chronologically comparable to that of PDLIM5 (threefold after 2 h in Figure S7A); and (4) RGS3 mRNA expression was upregulated in the CA1 region of the fasting mouse for 48 h compared with that in the fed mouse (Figure S7B). However, the data on RGS3 knockout clones were unclear. The lack of a viable commercial antibody prevented us from performing the necessary validation studies in our models. Considering the above, the fact that several RGS proteins––such as RGS4––are crucial for achieving the physiologically relevant timing and extent of GPCR signaling,^84^ and that the physiological function of RGS3 is largely unknown, the present data suggest it is worth to examine the fundamental role of RGS3 and its relationship with PDLIM5 in cilia shortening in future studies.

Our subtractive analysis of RNA‐seq with MCH detected 536 genes in MCHR1‐expressing hRPE1 cells. Most of the identified genes are likely related to other signaling events elicited by MCHR1 activation. These include receptor internalization, Ca^2+^ mobilization, ERK phosphorylation, and inhibition of cyclic AMP accumulation. As a proof of concept, we used the two essential pathways for cilia shortening (Akt and JNK) to isolate and identify transcripts that are related to ciliary shortening, successfully singling out PDLIM5 and possibly RGS3 transcripts out of the total regulated transcriptome. In a future study, we intend to perform additional RNA‐seq analysis of MCH‐treated cells with and without inhibitors of Akt and JNK to efficiently narrow the candidate list of transcripts related to cilia shortening and uncover potential new processes. For example, shortening of the axoneme must be accompanied by an efficient reduction in the surface area of the ciliary membrane. Ciliary endocytosis utilizing the vesicular membrane at the base is involved in the removal of the ciliary membrane.^85^, ^86^ However, the endocytotic control of signaling via ciliary GPCR remains a fundamentally unanswered question. By targeting the determined pathways during RNA‐seq, we may be able to answer such questions as that. It is also important to identify the transcription factors that ultimately result in PDLIM5 and alpha‐actinin 1/4 mRNA upregulation. Moreover, additional studies are needed to determine the exact mechanism by which PDLIM5 and alpha‐actinin 1/4 facilitate ciliary dynamics via actin remodeling. Both of PDLIM5 and alpha‐actinin 1/4 lack a spatial relationship with cilia in our study, so how these proteins could influence cilia length? The actin cytoskeleton modulates ciliary dynamics through effects on both actin network remodeling and cilia‐targeted vesicle trafficking.^10^, ^30^, ^31^ Therefore, it could be possible, at least in part, that an enlarged actin meshwork caused by PDLIM5‐alpha‐actinin 1/4 complex does not allow the arrival of transport vesicles at the cilia base that is necessary to maintain cilia length of the right size. Alternatively, conducting a mutational analysis of PDLIM5 and alpha‐actinin 1/4 may suggest a yet unknown route, which seems to participate in the modulation of centrosome‐derived actin regulators such as Arp2/3 and myosin Va.^33^, ^34^, ^35^, ^36^ Overall, new screening strategies may allow us to investigate‐specific aspects of ciliary physiology.

**FIGURE 9:**
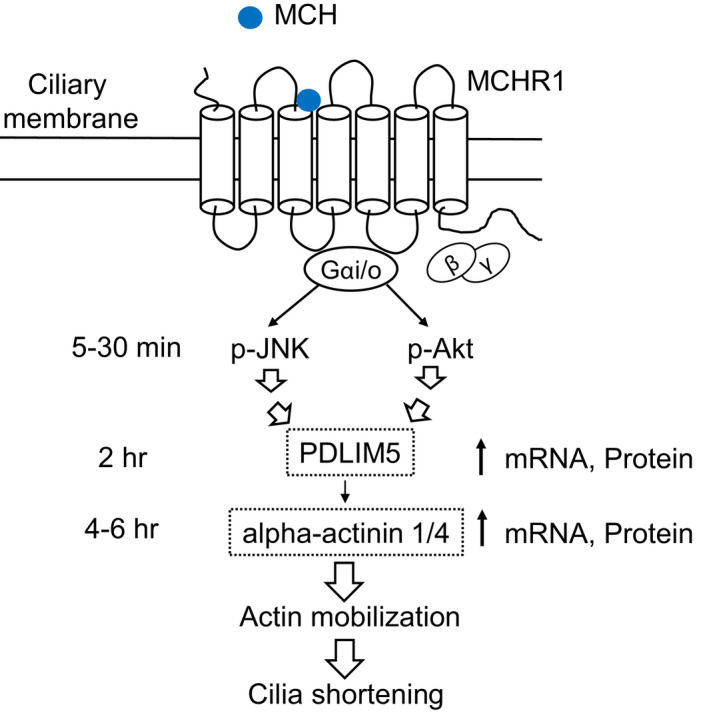
A model of MCH‐MCHR1 signaling in cilia length shortening. PDLIM5 is the most significant key factor for MCHR1‐mediated shortening of cilia length. Alpha‐actinin 1/4 as an F‐actin regulator by cross‐linking F‐actin is a crucial downstream target of the PDLIM5 signaling pathway that causes MCHR1‐induced cilia shortening. First, MCH binds to MCHR1 on the cilium, resulting in the activation of Gi/o‐Akt and Gi/o‐JNK to transiently increase PDLIM5 mRNA expression (treatment with MCH for 2 h). This leads to the upregulation of its protein products. Next, treatment with MCH for 4 and 6 h increases the expression of alpha‐actinin 1 and 4 mRNA and protein expression, which interact with PDLIM5. This upregulation is blocked by PDLIM5 knockdown, suggesting a link between alpha‐actinin 1/4 and PDLIM5. Alpha‐actinin 1/4 mRNA expression is not upregulated at early time points, indicating that upregulated PDLIM5 initially binds to the constitutive preexisting alpha‐actinin 1/4, which leads to the rearrangement of actin filaments (actin polymerization, actin bundling). The actin network participates in cilia lengthening through the effect on the long‐range intracellular trafficking of vesicles to the ciliary base.^8^, ^18^, ^19^ It is conceivable, therefore, that a larger actin meshwork elicited by the PDLIM5‐alpha‐actinin 1/4 complex might not allow the arrival of materials at the centrosome that are necessary for its maintenance, resulting in ciliary shortening

In conclusion, this study provides an important insight into ciliary MCHR1‐mediated cilia shortening elicited by PDLIM5 and its partner alpha‐actinin 1/4, both of which are actin‐related players (Figure 9). To further strengthen our findings, in addition to hRPE1 cells as representative cilia models, we also showed the underpinnings of a similar mechanism using endogenous MCHR1‐expressing hippocampal cells in rats and mice. This is particularly relevant when considering recent transcriptome‐wide association studies that show dysregulation in the expression of several GPCRs including MCHR1, in individuals with schizophrenia^87^ and the implication of single nucleotide polymorphisms in the PDLIM5 gene and susceptibility to neurological conditions, including schizophrenia.^77^, ^78^, ^79^ Thus, the study of PDLIM5‐based interaction networks focused on MCHR1‐bearing primary cilia will contribute to improving the understanding of mental illnesses and serve as a basis for developing potential therapies targeting the primary cilia.

## CONFLICT OF INTEREST

Declarations of interest: T. Shirao is CEO of AlzMed, Inc. No potential conflict of interest relevant to this article for Y. Kobayashi, S. Tomoshige, K. Imakado, Y. Sekino, N. Koganezawa, T. Miyamoto, and Y. Saito was reported.

## AUTHOR CONTRIBUTIONS

Y. Kobayashi designed the project, performed the experiments, analyzed and interpreted the data, and wrote the manuscript. S. Tomoshige and K. Imakado performed the experiments and analyzed, and interpreted the data. Y. Sekino, N. Koganezawa, and T. Shirao provided resources and interpreted the data. GB. Diniz interpreted the data and revised the manuscript accordingly. T. Miyamoto performed the experiments, provided the resources, interpreted the data, and revised the manuscript. Y. Saito directed and designed the project, performed the experiments, analyzed and interpreted the data, and wrote the manuscript. All authors approved the final manuscript.

## Supporting information

Fig S1‐S7

Table S1

Table S2

Table S3

Table S4

Table S5

Table S6

Table S7
