# Supplementary material for: Ciliary GPCR‐based transcriptome as a key regulator of cilia length control
Source: FASEB Bioadv. 2021 Jul 5;3(9):744–67. doi: 10.1096/fba.2021-00029 (PMC8409570; doi:10.1096/fba.2021-00029)
Supplement: Supplementary file 1 — Fig S1‐S7 [file FBA2-3-744-s003.pdf]

Supplemental Fig.1

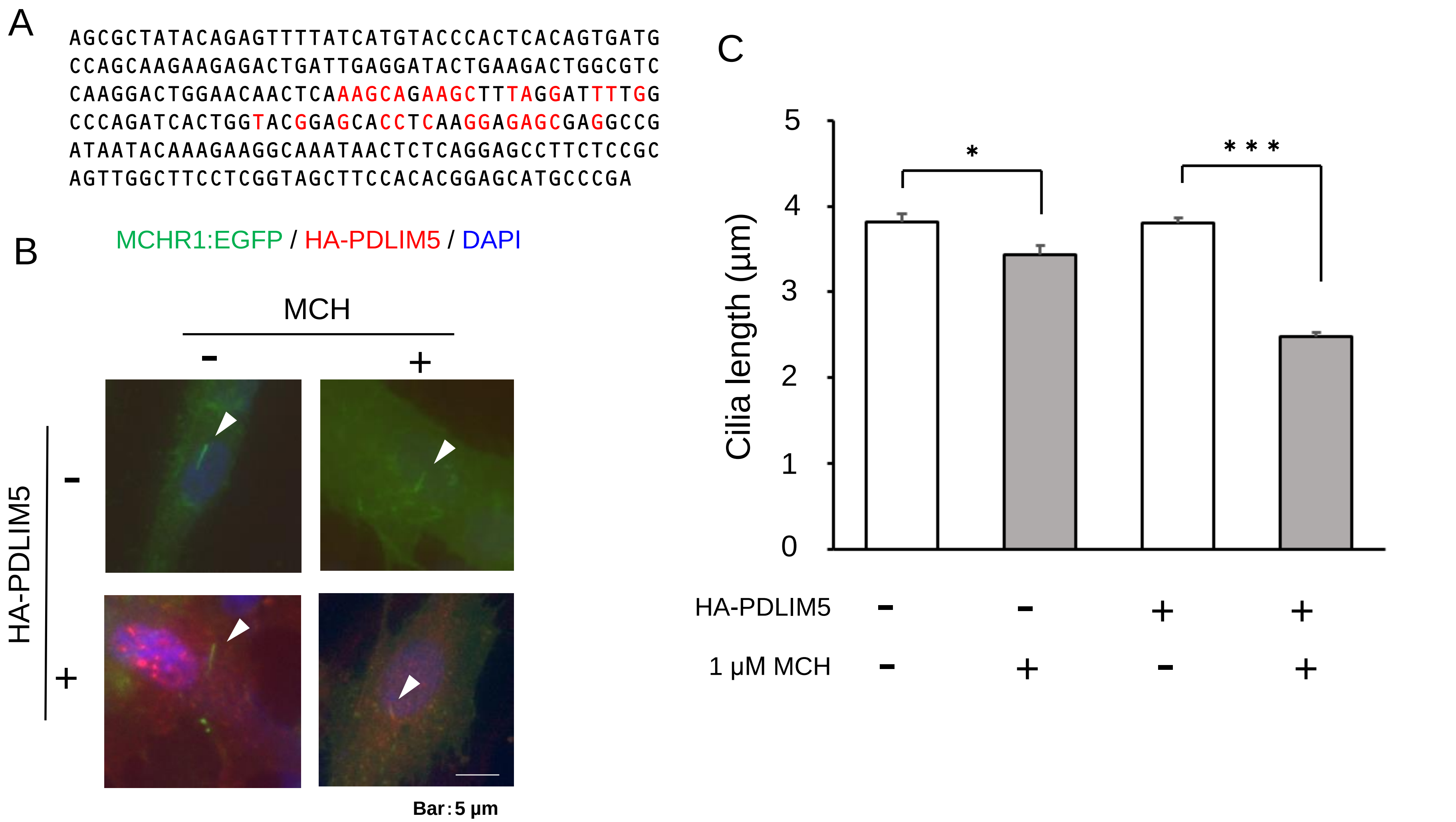

Supplemental Fig. 1 Exogenous PDLIM5 expression restores the impaired effect of MCH-induced response in PDLIM5 siRNA-transfected cells. (A) The gBlocks DNA fragment sequences of PDLIM5 containing silent mutations in the siRNA target regions (#1 and #2). Red letters are silent mutations. (B) Typical primary cilia images of MCH-treated and untreated PDLIM5 siRNA (#1 and #2) - transfected cells with and without HA-tagged PDLIM5, respectively. Arrowhead indicates the primary cilium. (C) Interference with the function of PDLIM5 reduces a similar magnitude of ciliary shortening after MCH exposure (p = 0.0128) to PDLIM5 siRNA-treated MCHR1:EGFP clone cells in Fig. 4A. On the other hand, exogenous PDLIM5 rescue (expression HA-PDLIM5 +) restores primary ciliary shortening in MCH stimulation to the same level as MCH-treated MCHR1:EGFP clone cells. The significance test compared with MCH is performed using a two-way analysis of variance and the Tukey–Kramer method (*$p < 0.05$, ***$p < 0.001$).

Supplemental Fig.2

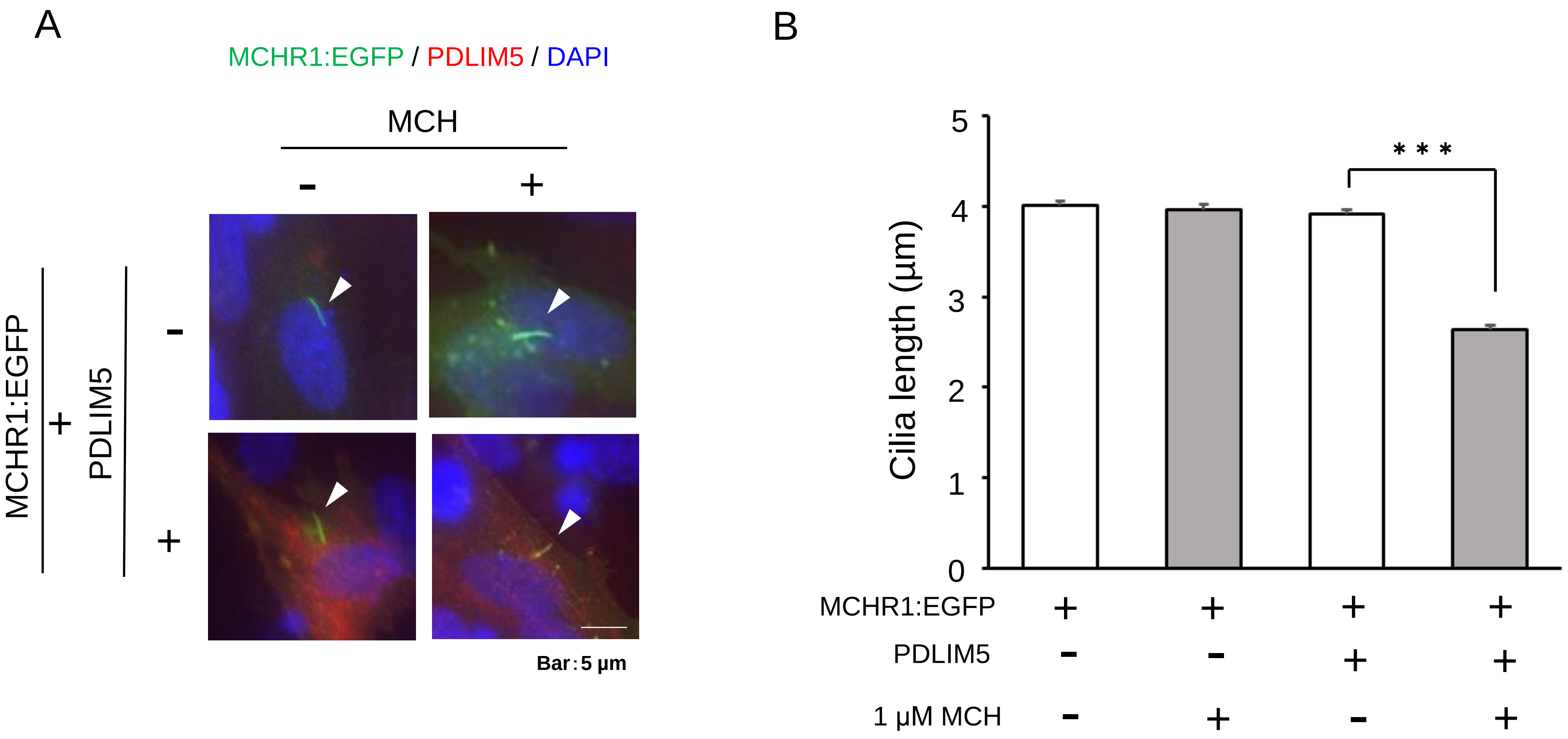

Supplemental Fig. 2 Exogenous PDLIM5 expression rescues the lack of MCH-induced response to MCH in PDLIM5 knockout cells. (A) Typical primary cilia images of MCH-treated or untreated PDLIM5 knockout cells with and without exogenous HA-tagged PDLIM5. Arrowhead indicates the primary cilium. (B) PDLIM5 knockout cells do not respond to MCH, while PDLIM5 knockout cells transfected with exogenous PDLIM5 restore MCH-induced ciliary shortening. The significance test compared with MCH is performed using a two-way analysis of variance and the Tukey–Kramer method (***$p < 0.001$).

Supplemental Fig.3

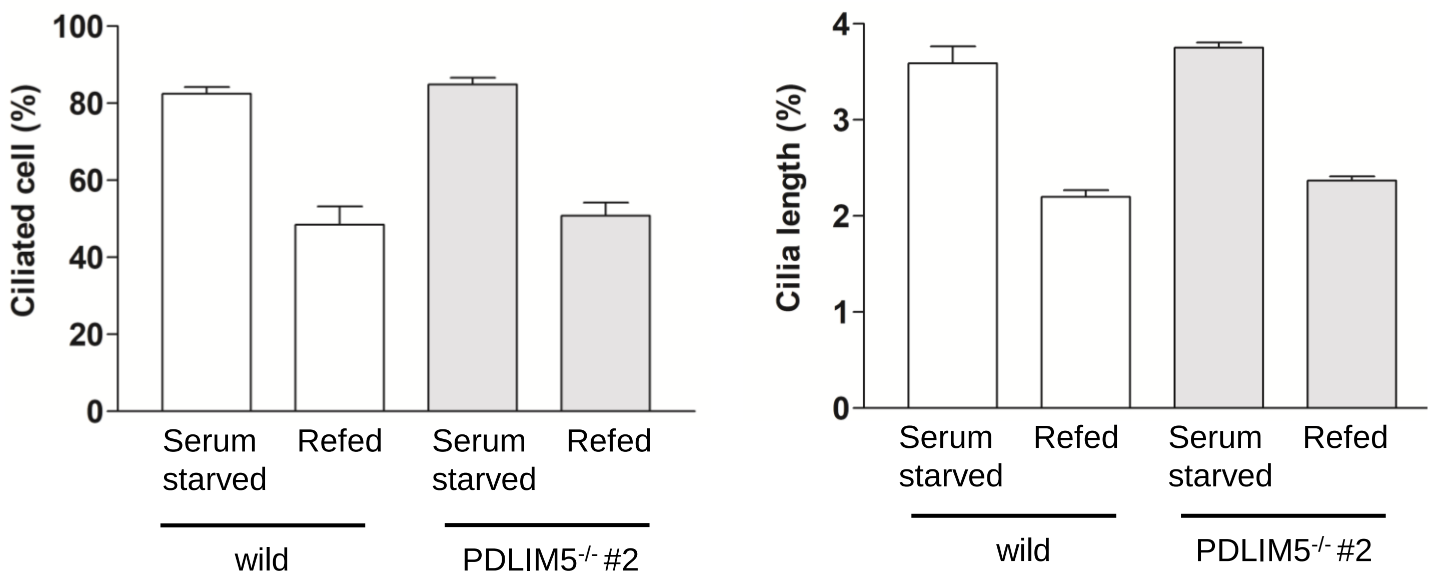

Supplemental Fig. 3 PDLIM5 knockout does not affect the basic signaling cascades of ciliogenesis following serum addition and starvation. The percentage of ciliated cells (left panel) is not significantly different between wild-type (wild) and PDLIM5 knockout cells (PDLIM5-/-) under serum-starved and refed conditions, respectively, as determined by Student's *t*-test. Similarly, cilia length (right panel) is not significantly different between wild and PDLIM5-/- cells under serum-starved and refed conditions, respectively, as determined by Student's *t*-test.

Supplemental Fig.4

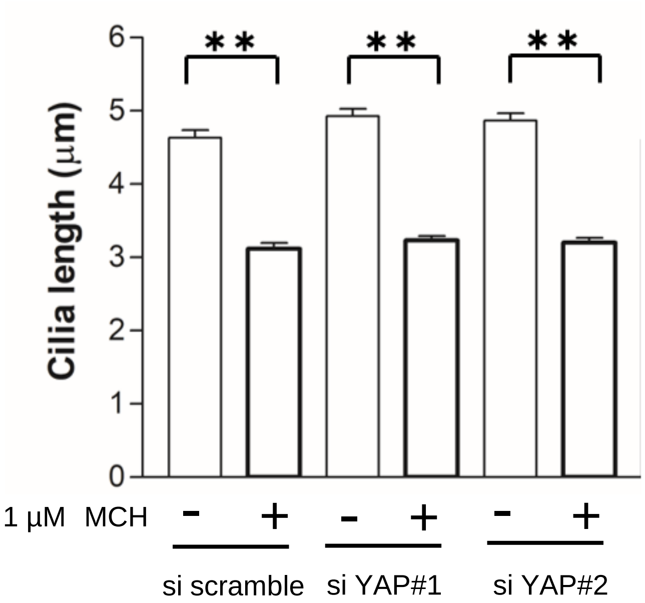

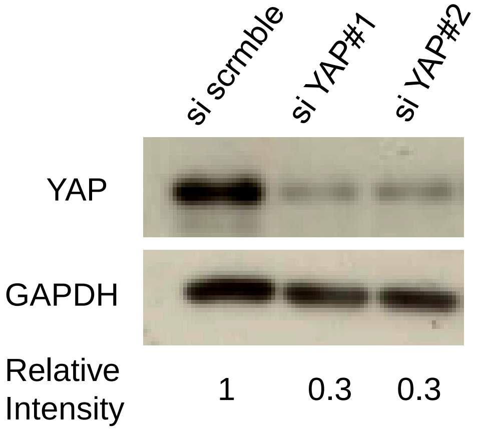

Supplemental Fig. 4 YAP knockdown does not affect the extent of cilia shortening through the MCH-MCHR1 interaction. Knockdown experiments using two independent YAP siRNAs does not affect cilia shortening mediated by MCH treatment for 6 hr in MCHR1:EGFP clone cells. The significance test compared with MCH is performed using a two-way analysis of variance and the Tukey–Kramer method (**$p < 0.01$) (left). The Western blotting analysis shows that YAP protein is reduced by 70% in MCHR1:EGFP treated with YAP siRNA (right).

Supplemental Fig.5

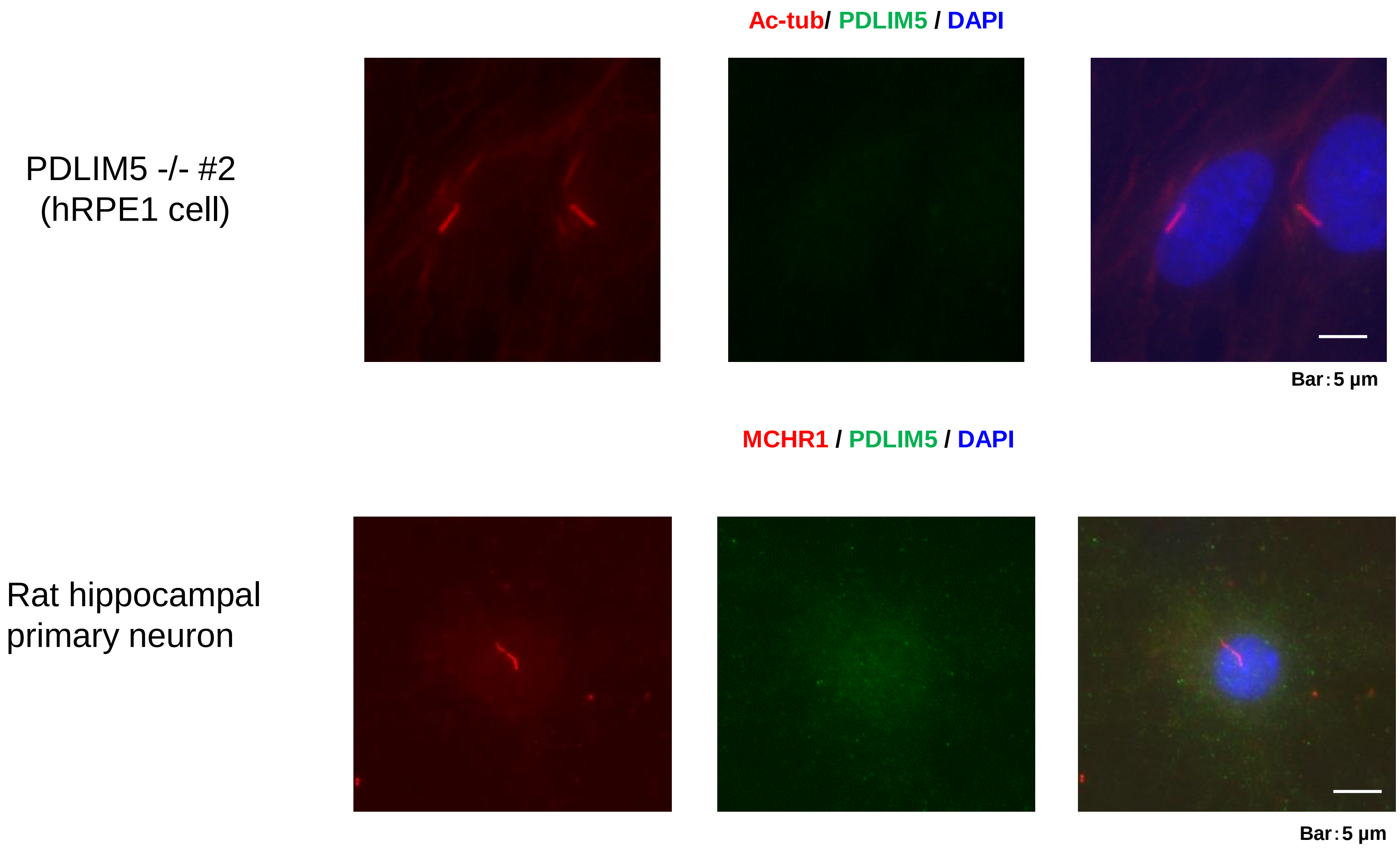

Supplemental Fig. 5 PDLIM5 immunostaining is not detected in PDLIM5 knockout cells and rat hippocampal primary neurons. The PDLIM5 antibody employed in this study is highly specific because immunostaining with the antibody is not observed in PDLIM5 knockout cells (PDLIM5-/-, upper panels). Using this validated antibody, PDLIM5-specific signals are not detected in somatic and neurites in primary cultures of 18 DIV (bottom panels).

Supplemental Fig.6

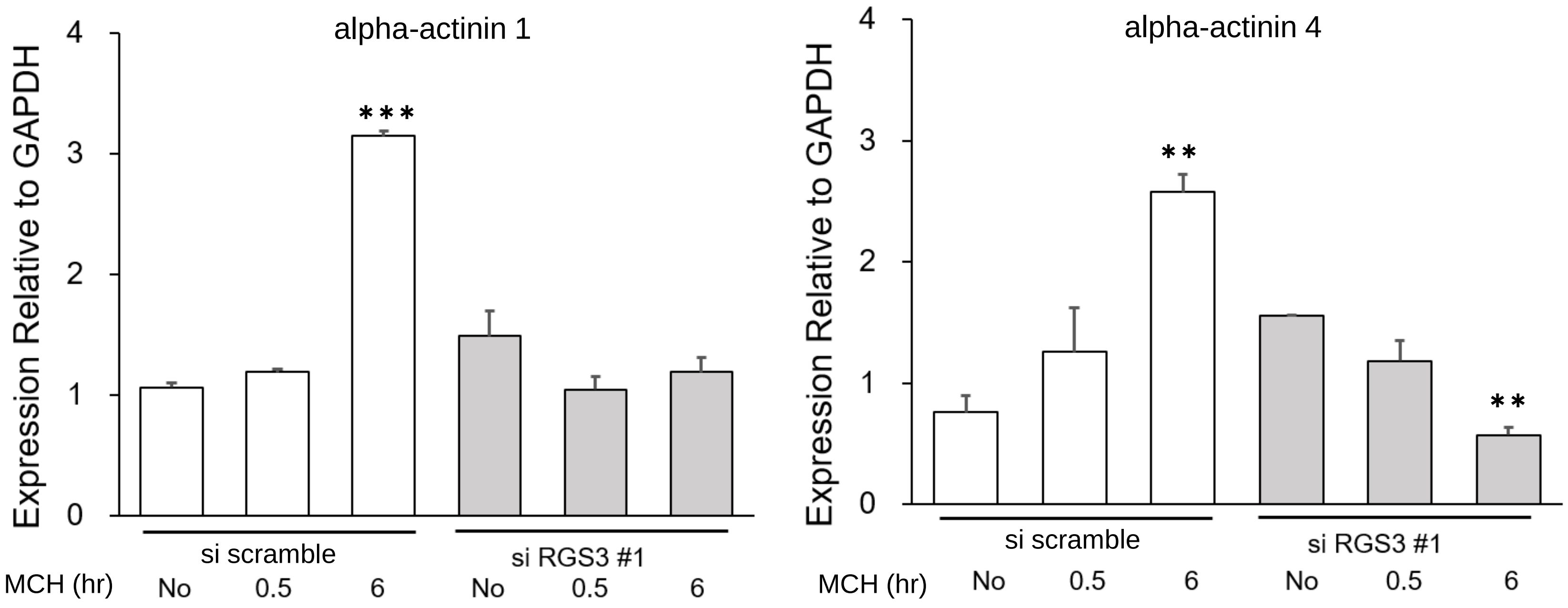

Supplemental Fig. 6 RGS3 knockdown significantly blocks the MCH-induced upregulation of alpha-actinin 1/4 in MCHR1:EGFP-expressing clone cells. In MCHR1:EGFP transfected with RGS3 siRNA, the behavior of the alpha-actinin 1/4 mRNA accompanied with MCH treatment is investigated using qRT-PCR. In the control scramble, both the alpha-actinin 1 and 4 mRNA levels significantly increases after 6 hr of MCH treatment. Meanwhile, each upregulation is significantly attenuated by RGS3 knockdown. Significance tests compared with the control (no MCH) in each group are performed using a two-way analysis of variance and the Tukey–Kramer method (** $p < 0.01$, *** $p < 0.001$).

Supplemental Fig.7

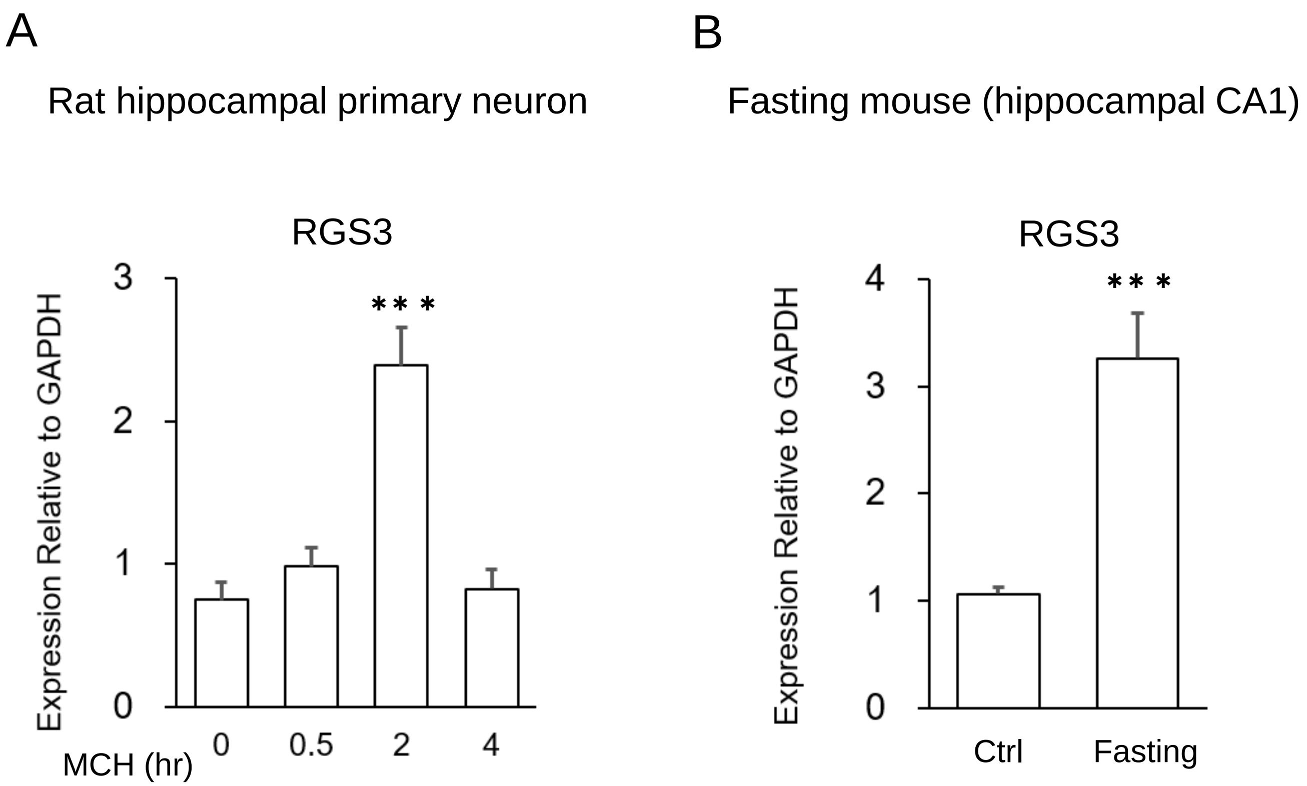

Supplemental Fig. 7 RGS3 mRNA expression is upregulated in MCH-treated cultured rat hippocampal neurons and hippocampal CA1 region of fasting mice. (A) Addition of MCH to cultured rat hippocampal neurons at 18 DIV causes a transient increase in RGS3 mRNA expression. The significance test for 0 hr is evaluated using the Tukey–Kramer method (****p* < 0.001). (B) Compared with fed mice, the mRNA expression of RGS3 is upregulated in the hippocampal CA1 region of the mouse after fasting for 48 hr by approximately threefold (Student *t*-test, ****p* < 0.001).
