# Supplementary material for: Ciliary GPCR‐based transcriptome as a key regulator of cilia length control
Source: FASEB Bioadv. 2021 Jul 5;3(9):744–67. doi: 10.1096/fba.2021-00029 (PMC8409570; doi:10.1096/fba.2021-00029)
Supplement: Supplementary file 2 — Table S1 [file FBA2-3-744-s005.pdf]

Supplemental Table 1. Primer sequences used for quantitative RT-PCR

Gene name	Primer sequence (5'-3')		Species	Experiment
	Forward	Reverse		
ATF3	cctctgcgctggaatcagtc	ttctttctcgtcgcctcttttt	Human	Fig.2, 3
ARC	agcgggacctgtaccagac	gcaggaaacgcttgagcttg	Human	Fig.2, 3
BMF	gggttattcgggatggtt	ccttcctgctagggtgtct	Human	Fig.2, 3
FOSB	gtgtgagcgcttctgcagc	ccaattcaacggctcgctt	Human	Fig.2, 3
MAFF	agagaggaaggtggcaggtc	ggataggggatccacagaca	Human	Fig.2, 3
PDLIM5	caccacaacctccatctccc	ccagcctgaccaacataggg	Human	Fig.2, 3, 6C
PRKAG2	ccctatcagtgggaatgcac	gctcatccaggttctgcttc	Human	Fig.2, 3
RAB3B	agatggtcccagtaatagatactc	gactgttctctaagtccctgtagt	Human	Fig.2, 3
RAB23	gaaagtagtagccgaagtgggagat	agtgcctcagcttcctcattctt	Human	Fig.2, 3
RGS2	ctgtgacctgccataaagactg	cagaccacctattcccttcttg	Human	Fig.2, 3
RGS3	acggagaaagcgagggtacg	gcacaccaaggacaacctgc	Human	Fig.2, 4A, 6C
RGS4	aacacaattcttcccacaacaa	ctgccagcccacattca	Human	Fig.2, 3
alpha-actinin 1	ggttatgatattggcaacgacc	gctgtatctgtgtcggctgt	Human	Fig.7 A, B, S6
alpha-actinin 4	gtcctgcttctaccacgctt	caggtgctcgttctcttggt	Human	Fig.7A, B, S6
GAPDH	tgagcttgacaaagtggtcg	gagcaccaggtggtctcc	Human	Fig.2, 3, 4A, 6C, 7A, B, S6
PDLIM5	agtctgagccagttgctgtc	aagggccgtggcactttatt	Rat	Fig.8A
alpha-actinin 1	tgaacgaattccgagcctcc	ggaacgtcactacccccaag	Rat	Fig.8A
alpha-actinin 4	ggcacagacctgagctgatt	ggctgtgttcacgatgtcct	Rat	Fig.8A
RGS3	aggatctgcagccatgaacc	gcagatggtgaagccaaagc	Rat	Fig.S7A
GAPDH	agtgccagcctcgtctcata	gactgtgccgttgaacttgc	Rat	Fig.8A, S7A
PDLIM5	tgcccactgcaaccaagtta	gccacattcaggagcaaagg	Mouse	Fig.8B
alpha-actinin 1	tgatattggcaacgaccccc	gatgaaggcctggaacgtca	Mouse	Fig.8B
alpha-actinin 4	cacaggcctgagctgattga	ggctgtgttcacgatgtcct	Mouse	Fig.8B
RGS3	tcctctggggaacttggatgt	tttttgctgggtgcgggttc	Mouse	Fig.S7B
GAPDH	agaaggtggtgaagcagggcatc	cgaaggtggaagagtgggagttg	Mouse	Fig.8B, S7B
