# Supplementary material for: Ciliary GPCR‐based transcriptome as a key regulator of cilia length control
Source: FASEB Bioadv. 2021 Jul 5;3(9):744–67. doi: 10.1096/fba.2021-00029 (PMC8409570; doi:10.1096/fba.2021-00029)
Supplement: Supplementary file 3 — Table S2 [file FBA2-3-744-s008.pdf]

Supplemental Table 2

Effects of siRNA transfection on cilia generation in MCHR1:EGFP-expressing clone cells

siRNA	MCHR1/DAPI (%)
scramble	68.20 ± 2.21
RGS3 #1	72.28 ± 1.96
PDLIM #1	71.10 ± 3.54
PDLIM #2	69.50 ± 1.47
alpha-actinin 1 #2	68.48 ± 2.81
alpha-actinin #1	68.61 ± 1.53
alpha-actinin 1 #2 + alpha-actinin 4 #1	73.44 ± 2.79

For siRNA, hRPE1 clone cells expressing MCHR1:EGFP were seeded, cultured for 18 hr and transfected with 20 pmol siRNA using Lipofectamine RNAiMAX. Transfected cells were cultured in normal growth medium for 18 hr, then serum-starved for 24 hr, fixed and stained with DAPI in PBS. The percentage in the MCHR1-positive cilia per cell were determined under a fluorescence microscope. The data represent means ± SEM of two independent experiments (> 100 cells per experiment). The significance test for scramble was evaluated by the Tukey–Kramer method, but no significant difference was detected between all groups.
