# Supplementary material for: Ciliary GPCR‐based transcriptome as a key regulator of cilia length control
Source: FASEB Bioadv. 2021 Jul 5;3(9):744–67. doi: 10.1096/fba.2021-00029 (PMC8409570; doi:10.1096/fba.2021-00029)
Supplement: Supplementary file 4 — Table S3 [file FBA2-3-744-s004.pdf]

Supplemental Table 3. Target genomic sites in hRPE1 cells for the CRISPR-ObLiGaRe-mediated gene knockout

Target genes	Target exons	gRNA and target PAM (5'-NGG-3') sequence	Genotyping primer pairs
PDLIM5	Exon 3	'5'-ATTGATGGAATAAATGCACA(AGG)-3'	5'-CTTTTTCAAAGAGTGACACTCCTAATG-3'
			5'-CCTGAAATGATGGTAGCAGAAACTGAGTC-3'
RGS3	Exon 14	5'-AGCTCTGATGGGCTGCTGCT(CGG)-3	5'-CAGTCACCCCCCAACAAACGGGAGAAG-3'
			5'-AGGCAGGGTGTGCTGGCCCCCGCGCTTG-3'
