# Supplementary material for: Ciliary GPCR‐based transcriptome as a key regulator of cilia length control
Source: FASEB Bioadv. 2021 Jul 5;3(9):744–67. doi: 10.1096/fba.2021-00029 (PMC8409570; doi:10.1096/fba.2021-00029)
Supplement: Supplementary file 5 — Table S4 [file FBA2-3-744-s002.pdf]

Supplemental Table 4. Genotypes of the edited hRPE1 cell lines using the CRISPR-ObLiGaRe method

Cell line ID	Target exon	Allele 1 (ObLiGaRe-mediated *NeoR* cassette integration)	Allele 2 (CRISPR/Cas9-mediated indel mutations)	
			DNA sequence alteration	Amino acid alteration
PDLIM5 -/- #1	Exon 3	NeoR (+): Forward integration	CDS 172 ins C	Q59fs
PDLIM5 -/- #2	Exon 3	NeoR (+): Forward integration	CDS 172 del A	Q59fs
RGS3 -/- #1	Exon 14	NeoR (+): Forward integration	CDS ins T	L436fs
RGS3 -/- #2	Exon 14	NeoR (+): Forward integration	CDS del T	L435fs
RGS3 -/- #3	Exon 14	NeoR (+): Forward integration	CDS del T	L435fs

CDS: coding sequence, ins: base insertion, del: base deletion, fs: frameshift
