# Supplementary material for: Ciliary GPCR‐based transcriptome as a key regulator of cilia length control
Source: FASEB Bioadv. 2021 Jul 5;3(9):744–67. doi: 10.1096/fba.2021-00029 (PMC8409570; doi:10.1096/fba.2021-00029)
Supplement: Supplementary file 6 — Table S5 [file FBA2-3-744-s006.pdf]

Supplemental Table 5. Comprehensive search for genes that fluctuate with MCH 2 hr stimulation using RNA-Seq analysis

Differences in gene expression were investigated between untreated and treated MCHR1:EGFP clone cells with 1 μM MCH for 2 hr.
We have listed all genes that fluctuate more than twice with the normalized value (FPKM).

Gene_ID	Transcript_ID	Gene_Symbol	MCH_2h/No MCH fold change	No MCH_FPKM	MCH 2hr_FPKM	Gene_Description
3576	NM_000584	IL8	206.210394	0.409470	291.263000	interleukin 8
22943	NM_012242	DKK1	79.530577	2.426680	270.983000	dickkopf WNT signaling pathway inhibitor 1
2354	NM_001114171	FOSB	44.748290	0.856820	81.794000	FBJ murine osteosarcoma viral oncogene homolog B
3164	NM_002135,NM	NR4A1	41.774246	1.789611	113.713040	nuclear receptor subfamily 4 group A member 1 isoform 3
2921	NM_002090	CXCL3	38.186240	0.067632	39.827500	chemokine (C-X-C motif) ligand 3
1839	NM_001945	HBEGF	23.085688	2.774830	85.833700	heparin-binding EGF-like growth factor
3589	NM_000641,NM	IL11	22.611907	5.163180	140.385587	interleukin 11
5997	NM_002923	RGS2	22.549238	8.094880	211.071000	regulator of G-protein signaling 2, 24kDa
5054	NM_000602	SERPINE1	21.533125	46.751300	966.923000	serpin peptidase inhibitor, clade E (nexin, plasminogen activator inhibitor type 1), member 1
79094	NM_024111,NM	CHAC1	19.276818	0.905190	35.404270	ChaC, cation transport regulator homolog 1 (E. coli)
4929	NM_006186	NR4A2	18.672813	1.234410	39.889800	nuclear receptor subfamily 4, group A, member 2
5744	NM_002820,NM	PTHLH	18.369212	1.751631	49.140890	parathyroid hormone-like hormone
23764	NM_001161573	MAFF	17.535372	3.659480	80.821309	v-maf avian musculoaponeurotic fibrosarcoma oncogene homolog F
8013	NM_173199,NM	NR4A3	17.463629	0.150425	19.350593	nuclear receptor subfamily 4, group A, member 3
5743	NM_000963	PTGS2	16.963751	0.608815	26.316400	prostaglandin-endoperoxide synthase 2 (prostaglandin G/H synthase and cyclooxygenase)
467	NM_001206488	ATF3	15.942387	0.848600	28.634464	activating transcription factor 3
3569	NM_000600	IL6	14.143290	1.245210	30.522900	interleukin 6 (interferon, beta 2)
1490	NM_001901	CTGF	13.829366	132.629000	1736.950000	connective tissue growth factor
1437	NM_000758	CSF2	13.010648	0.108979	13.601200	colony stimulating factor 2 (granulocyte-macrophage)
9510	NM_006988	ADAMTS1	12.845903	0.902951	23.595100	ADAM metallopeptidase with thrombospondin type 1 motif, 1
3976	NM_001257135	LIF	12.787567	38.511420	502.507000	leukemia inhibitory factor
1649	NM_004083,NM	DDIT3	12.480743	28.263059	369.159979	DNA-damage-inducible transcript 3
1960	NM_004430,NM	EGR3	12.138981	2.168396	37.037940	early growth response 3
6446	NM_001291995	SGK1	12.073145	16.192423	214.218664	serine/threonine-protein kinase Sgk1 isoform 5
5055	NM_002575,NM	SERPINB2	12.035473	0.494384	17.076612	serpin peptidase inhibitor, clade B (ovalbumin), member 2
84879	NM_001287809	MFSD2A	11.655472	0.492659	16.573776	major facilitator superfamily domain containing 2A
2920	NM_002089	CXCL2	11.134536	0.225108	12.740100	chemokine (C-X-C motif) ligand 2
374	NM_001657	AREG	11.102665	0.285098	13.379300	amphiregulin
1827	NM_004414,NM	RCAN1	10.484394	7.085596	85.322437	regulator of calcineurin 1
2919	NR_046035,NM	CXCL1	10.391142	0.066519	10.293917	chemokine (C-X-C motif) ligand 1 (melanoma growth stimulating activity, alpha)
23529	NM_001166212	CLCF1	9.567466	11.558440	121.291400	cardiotrophin-like cytokine factor 1
1847	NM_004419	DUSP5	9.465188	7.287400	78.411900	dual specificity phosphatase 5
8061	NM_001300855	FOSL1	8.936252	24.362297	230.994520	fos-related antigen 1 isoform 2
1958	NM_001964	EGR1	8.818309	10.018900	98.944600	early growth response 1
9734	NM_001204148	HDAC9	8.752576	0.480920	12.031765	histone deacetylase 9
5021	NM_000916	OXTR	8.694670	0.494708	12.092000	oxytocin receptor
5999	NM_001113380	RGS4	8.590743	52.149635	457.092857	regulator of G-protein signaling 4
9709	NM_001272103	HERPUD1	8.578337	37.842291	327.915350	homocysteine-inducible, endoplasmic reticulum stress-inducible, ubiquitin-like domain member 1
23753	NM_022044	SDF2L1	8.237888	32.659900	284.751000	stromal cell-derived factor 2-like 1
3624	NM_002192	INHBA	8.152665	1.399620	18.472800	inhibin, beta A
23237	NM_015193	ARC	8.111048	0.011863	7.261720	activity-regulated cytoskeleton-associated protein
26585	NM_013372,NM	GREM1	8.002508	15.081553	131.707765	gremlin 1, DAN family BMP antagonist
3491	NM_001554	CYR61	7.681017	86.462400	667.970000	cysteine-rich, angiogenic inducer, 61
4783	NM_001290000	NFIL3	7.644255	3.121447	30.651902	nuclear factor interleukin-3-regulated protein
2152	NM_001178096	F3	7.263814	14.685218	115.687670	coagulation factor III (thromboplastin, tissue factor)
5971	NM_006509	RELB	7.207126	4.123570	36.101900	v-rel avian reticuloendotheliosis viral oncogene homolog B
4739	NR_073131,NM	NEDD9	6.908885	0.470234	9.200016	neural precursor cell expressed, developmentally down-regulated 9
2355	NM_005253	FOSL2	6.839421	5.107760	41.103200	FOS-like antigen 2
29950	NM_013376	SERTAD1	6.751846	17.219900	124.335000	SERTA domain containing 1
10468	NM_013409,NM	FST	6.740761	6.438144	49.872820	follistatin
5292	NM_001243186	PIM1	6.600993	4.194170	33.500200	pim-1 oncogene
29970	NM_001197107	SCHIP1	6.556669	6.671912	50.125406	schwannomin interacting protein 1
627	NM_170735,NM	BDNF	6.479532	3.658428	29.316536	brain-derived neurotrophic factor
4254	NM_003994,NM	KITLG	6.307088	1.388442	14.023110	KIT ligand
51330	NM_016639	TNFRSF12A	6.274170	86.582800	556.982000	tumor necrosis factor receptor superfamily, member 12A
25819	NM_012118	CCRN4L	6.272030	0.784044	10.249800	CCR4 carbon catabolite repression 4-like (S. cerevisiae)
221749	NM_183373	PXDC1	5.989106	13.260400	86.383100	PX domain containing 1
23034	NM_015589,NM	SAMD4A	5.852941	0.724544	9.112004	sterile alpha motif domain containing 4A
54206	NM_018948	ERRFI1	5.797733	48.821600	294.943000	ERBB receptor feedback inhibitor 1
5606	NM_145109,NM	MAP2K3	5.795559	24.715930	151.925200	mitogen-activated protein kinase kinase 3
6236	NM_001128850	RRAD	5.784196	3.448698	24.897270	Ras-related associated with diabetes
6385	NM_002999	SDC4	5.724272	38.363700	227.090000	syndecan 4
1052	NM_005195	CEBPD	5.716662	2.549160	19.393500	CCAAT/enhancer binding protein (C/EBP), delta
168455	NM_175884	CCDC71L	5.631232	6.488830	41.646300	coiled-coil domain containing 71-like
10797	NR_027405,NM	MTHFD2	5.569903	8.348796	52.850820	methylenetetrahydrofolate dehydrogenase (NADP+ dependent) 2, methenyltetrahydrofolate cyclohydrolase
688	NM_001286818	KLF5	5.374345	5.200113	32.945450	Kruppel-like factor 5 (intestinal)
8140	NM_003486	SLC7A5	5.349796	5.822730	36.104100	solute carrier family 7 (amino acid transporter light chain, L system), member 5
100505385	NM_001197113	IQCJ-SCHIP1	5.345915	4.120899	26.642109	IQCJ-SCHIP1 readthrough
51421	NM_001278683	AMOTL2	5.326706	3.392151	22.690034	angiomotin like 2
2627	NM_005257	GATA6	5.294175	7.376110	44.058100	GATA binding protein 6
27063	NM_014391	ANKRD1	5.290089	11.428000	67.018500	ankyrin repeat domain 1 (cardiac muscle)
25907	NM_015444	TMEM158	5.140652	15.167300	84.601100	transmembrane protein 158 (gene/pseudogene)
92126	NM_032160	DSEL	5.117670	1.530630	11.886200	dermatan sulfate epimerase-like
8744	NM_003811	TNFSF9	5.097214	0.841045	8.383260	tumor necrosis factor (ligand) superfamily, member 9
2633	NM_002053	GBP1	5.034669	3.775390	23.231800	guanylate binding protein 1, interferon-inducible
10370	NM_001168389	CITED2	5.027929	54.572485	291.887839	Cbp/p300-interacting transactivator, with Glu/Asp-rich carboxy-terminal domain, 2
57801	NM_021170,NM	HES4	5.024236	3.669380	22.714890	hes family bHLH transcription factor 4
8870	NM_003897	IER3	5.018863	162.020000	804.470000	immediate early response 3
103504731	NR_128711	MIR3648-2	4.957453	1.289610	10.318500	microRNA 3648-2
65059	NM_203365,NM	RAPH1	4.953577	1.407644	10.894264	Ras association (RalGDS/AF-6) and pleckstrin homology domains 1
4773	NM_001258292	NFATC2	4.935536	5.218789	30.465170	nuclear factor of activated T-cells, cytoplasmic, calcineurin-dependent 2
970	NM_001252	CD70	4.924904	2.291660	15.174800	CD70 molecule
7358	NM_001184701	UGDH	4.923208	18.979900	100.195510	UDP-glucose 6-dehydrogenase
23012	NM_015000	STK38L	4.890552	7.624140	41.947100	serine/threonine kinase 38 like
2113	NM_001162422	ETS1	4.887448	5.912298	33.347756	v-ets avian erythroblastosis virus E26 oncogene homolog 1
387763	NM_001145033	C11orf96	4.714140	0.085942	4.083430	chromosome 11 open reading frame 96
91663	NM_001290189	MYADM	4.687273	30.517877	150.290615	myeloid-associated differentiation marker
4189	NM_012328	DNAJB9	4.629995	13.732900	69.254600	DnaJ (Hsp40) homolog, subfamily B, member 9
56911	NM_001286623	MAP3K7CL	4.629764	1.157793	8.963609	MAP3K7 C-terminal like
79669	NM_001171747	C3orf52	4.596513	2.174499	13.561720	chromosome 3 open reading frame 52
28984	NM_014059	RGCC	4.575123	0.567379	6.133760	regulator of cell cycle
2012	NM_001423	EMP1	4.558229	29.921900	143.519000	epithelial membrane protein 1
27	NM_001168239	ABL2	4.519334	1.580048	10.621850	c-abl oncogene 2, non-receptor tyrosine kinase
7280	NM_001069	TUBB2A	4.445119	8.581170	42.584700	tubulin, beta 2A class IIa
389136	NM_016206	VGLL3	4.442821	0.549176	5.863490	vestigial like 3 (Drosophila)
1848	NM_022652,NM	DUSP6	4.399032	12.676070	62.155500	dual specificity phosphatase 6
11080	NM_007034	DNAJB4	4.392583	4.998310	25.806200	DnaJ (Hsp40) homolog, subfamily B, member 4
51339	NR_046093,NM	DACT1	4.360378	0.413098	5.097390	dishevelled-binding antagonist of beta-catenin 1
2827	NM_005281	GPR3	4.355241	0.254794	4.388860	G protein-coupled receptor 3
9076	NM_021101	CLDN1	4.273998	0.030101	3.355460	claudin 1
8877	NM_021972,NM	SPHK1	4.247172	34.937660	154.519850	sphingosine kinase 1
415116	NM_001001852	PIM3	4.235635	8.483870	40.046200	pim-3 oncogene
9314	NM_004235	KLF4	4.212809	1.407420	9.144370	Kruppel-like factor 4 (gut)
2296	NM_001453	FOXC1	4.205722	0.984202	7.261690	forkhead box C1
3949	NM_001195798	LDLR	4.154323	7.733734	36.122600	low density lipoprotein receptor
5806	NM_002852	PTX3	4.148294	36.463800	157.883000	pentraxin 3, long
49854	NM_020727,NM	ZBTB21	4.146800	0.504761	5.198468	zinc finger and BTB domain containing 21
8482	NM_001146029	SEMA7A	4.133373	21.741344	95.345580	semaphorin 7A, GPI membrane anchor (John Milton Hagen blood group)
1959	NM_001136179	EGR2	4.102375	0.263601	4.118043	early growth response 2
11259	NM_001042459	FILIP1L	4.028813	0.582500	5.346483	filamin A interacting protein 1-like
23645	NM_014330	PPP1R15A	4.027953	24.794100	104.784000	protein phosphatase 1, regulatory subunit 15A
5154	NM_002607,NM	PDGFA	4.016520	2.580370	13.463925	platelet-derived growth factor alpha polypeptide
30844	NM_139265	EHD4	3.980972	2.469090	12.885700	EH-domain containing 4
11072	NM_007026	DUSP14	3.971526	16.635600	71.103600	dual specificity phosphatase 14
81606	NM_030915	LBH	3.895978	16.715600	69.904600	limb bud and heart development
1407	NM_004075	CRY1	3.890194	1.423340	8.415910	cryptochrome 1 (photolyase-like)

5271	NM_198833,NM	SERPINB8	3.874984	3.933264	18.296388	serpin peptidase inhibitor, clade B (ovalbumin), member 8
114789	NR_049766,NM	SLC25A25	3.870788	2.872602	14.037253	solute carrier family 25 (mitochondrial carrier; phosphate carrier), member 25
2353	NM_005252	FOS	3.869099	1.112720	7.069710	FBJ murine osteosarcoma viral oncogene homolog
79003	NM_00125822C	MIS12	3.842150	7.171338	31.030810	MIS12 kinetochore complex component
91319	NM_001002862	DERL3	3.816791	0.865091	6.068060	derlin 3
157506	NM_172037	RDH10	3.815235	23.463700	94.566400	retinol dehydrogenase 10 (all-trans)
112597	NR_024206,NR	LINC00152	3.809953	144.588378	557.515140	long intergenic non-protein coding RNA 152
9497	NM_003615,NM	SLC4A7	3.790847	2.190391	11.029734	solute carrier family 4, sodium bicarbonate cotransporter, member 7
6541	NM_003045	SLC7A1	3.770744	2.313390	11.439000	solute carrier family 7 (cationic amino acid transporter, y+ system), member 1
285958	NR_003697	SNHG15	3.756155	8.782650	36.666500	small nucleolar RNA host gene 15 (non-protein coding)
3422	NM_004508	IDI1	3.751519	19.431700	78.250100	isopentenyl-diphosphate delta isomerase 1
204010	NR_026825	RPSAP52	3.740900	0.861850	5.907100	ribosomal protein SA pseudogene 52
84444	NM_032482	DOT1L	3.713970	2.699770	12.790000	DOT1-like histone H3K79 methyltransferase
59274	NM_022566	MESDC1	3.707328	2.767650	13.051500	mesoderm development candidate 1
2318	NM_001127487	FLNC	3.692982	13.040070	52.439400	filamin C, gamma
147040	NM_001002914	KCTD11	3.689575	6.327040	26.582600	potassium channel tetramerization domain containing 11
571	NM_206866,NR	BACH1	3.677583	4.092250	17.822161	BTB and CNC homology 1, basic leucine zipper transcription factor 1
10395	NM_024767,NM	DLC1	3.617025	7.340787	29.903280	deleted in liver cancer 1
1054	NM_001806,NM	CEBPG	3.611663	3.318640	14.633130	CCAAT/enhancer binding protein (C/EBP), gamma
359845	NM_182705	FAM101B	3.609423	7.462790	30.319900	family with sequence similarity 101, member B
8553	NM_003670	BHLHE40	3.604473	11.447300	45.160400	basic helix-loop-helix family, member e40
3157	NM_002130,NM	HMGCS1	3.541658	6.191090	25.074400	3-hydroxy-3-methylglutaryl-CoA synthase 1 (soluble)
284207	NM_001004431	METRNL	3.520607	11.958900	45.956300	meteorin, glial cell differentiation regulator-like
124044	NM_152339	SPATA2L	3.497960	5.948490	23.906700	spermatogenesis associated 2-like
347733	NM_178012	TUBB2B	3.481533	3.906740	16.303400	tubulin, beta 2B class IIb
5998	NM_001282922	RGS3	3.479723	64.295154	232.499714	regulator of G-protein signaling 3
84617	NM_032525	TUBB6	3.469150	38.006100	137.107000	tubulin, beta 6 class V
9792	NM_014755	SERTAD2	3.462880	2.952900	12.784700	SERTA domain containing 2
29126	NM_014143,NR	CD274	3.453875	0.605001	4.442660	CD274 molecule
3638	NM_005542,NM	INSIG1	3.444511	15.567955	58.991415	insulin induced gene 1
64651	NM_033027	CSRNP1	3.436285	6.800150	26.386500	cysteine-serine-rich nuclear protein 1
4772	NM_001278669	NFATC1	3.422788	0.164329	2.909116	nuclear factor of activated T-cells, cytoplasmic, calcineurin-dependent 1
9242	NM_005098	MSC	3.416463	5.195600	20.826000	musculin
84981	NR_028503,NR	MIR22HG	3.406064	19.798620	72.377938	MIR22 host gene (non-protein coding)
80117	NM_025047	ARL14	3.365291	0.089053	2.602770	ADP-ribosylation factor-like 14
23243	NM_015199,NM	ANKRD28	3.360915	8.982346	33.511594	ankyrin repeat domain 28
5187	NM_002616	PER1	3.357251	1.101540	5.974580	period circadian clock 1
1266	NM_001839,NM	CNN3	3.341335	95.061587	318.051990	calponin 3, acidic
166929	NM_001136258	SGMS2	3.336207	1.715845	8.013534	sphingomyelin synthase 2
80271	NM_025194	ITPKC	3.326328	6.555910	24.636000	inositol-trisphosphate 3-kinase C
60673	NM_001098673	C12orf44	3.319784	43.144900	148.569400	chromosome 12 open reading frame 44
54908	NM_017785	SPDL1	3.319556	2.535280	10.740900	spindle apparatus coiled-coil protein 1
5329	NM_001005376	PLAUR	3.311103	71.527290	244.264040	urokinase plasminogen activator surface receptor isoform 4 precursor
81565	NM_030808,NM	NDEL1	3.300570	12.205460	43.743600	nudE neurodevelopment protein 1-like 1
4616	NM_015675	GADD45B	3.292075	40.808800	139.021000	growth arrest and DNA-damage-inducible, beta
9749	NM_001100166	PHACTR2	3.283341	2.228955	9.645061	phosphatase and actin regulator 2
83604	NM_031442	TMEM47	3.281357	2.999530	12.198800	transmembrane protein 47
65983	NM_023927,NM	GRAMD3	3.234003	4.795439	18.022926	GRAM domain containing 3
85480	NM_138551,NR	TSLP	3.225579	2.431690	10.185096	thymic stromal lymphopoietin
6722	NM_003131,NM	SRF	3.213428	12.705418	44.315920	serum response factor isoform 2
24147	NM_014344	FJX1	3.209269	0.930038	5.110990	four jointed box 1 (Drosophila)
90637	NM_182491	ZFAND2A	3.207893	22.180300	75.535900	zinc finger, AN1-type domain 2A
25822	NM_001135004	DNAJB5	3.196185	2.203108	9.255539	DnaJ (Hsp40) homolog, subfamily B, member 5
9590	NM_144497,NM	AKAP12	3.188833	8.559954	30.550350	A kinase (PRKA) anchor protein 12
157313	NM_152562	CDCA2	3.185494	0.268928	2.962420	cell division cycle associated 2
10253	NM_005842	SPRY2	3.162050	42.911200	140.358000	sprouty homolog 2 (Drosophila)
3156	NM_000859,NM	HMGCR	3.146958	7.868220	27.763800	3-hydroxy-3-methylglutaryl-CoA reductase
3777	NM_002246	KCNK3	3.144547	0.406218	3.326230	potassium channel, subfamily K, member 3
3309	NM_005347	HSPA5	3.127249	267.246000	818.325000	heat shock 70kDa protein 5 (glucose-regulated protein, 78kDa)
3475	NM_001197079	IFRD1	3.104754	7.577698	26.298172	n/a
1843	NM_004417	DUSP1	3.099801	10.547500	35.620000	dual specificity phosphatase 1
10272	NM_005860	FSTL3	3.098479	8.650930	30.061200	follistatin-like 3 (secreted glycoprotein)
84627	NM_001127464	ZNF469	3.084865	0.521468	3.588160	zinc finger protein 469
9655	NM_144949,NM	SOCS5	3.071937	8.326570	28.769590	suppressor of cytokine signaling 5
92162	NM_203411	TMEM88	3.070316	1.566540	6.763000	transmembrane protein 88
23516	NM_015359,NM	SLC39A14	3.067694	11.995288	39.624309	solute carrier family 39 (zinc transporter), member 14
6675	NM_003115	UAP1	3.041571	22.213700	71.965700	UDP-N-acteylglucosamine pyrophosphorylase 1
7975	NM_002360	MAFK	3.035608	3.593820	13.020400	v-maf avian musculoaponeurotic fibrosarcoma oncogene homolog K
23670	NM_001135820	TMEM2	3.025694	1.498249	6.433317	transmembrane protein 2
2250	NM_001291812	FGF5	3.012131	5.873743	20.380451	fibroblast growth factor 5 isoform 3
6648	NM_001024466	SOD2	2.975950	16.485640	52.658300	superoxide dismutase 2, mitochondrial
440	NM_133436,NM	ASNS	2.967966	7.999108	26.553213	asparagine synthetase (glutamine-hydrolyzing)
4790	NM_003998,NM	NFKB1	2.962928	5.071180	17.318740	nuclear factor of kappa light polypeptide gene enhancer in B-cells 1
132864	NM_001177384	CPEB2	2.947697	1.340684	5.794155	cytoplasmic polyadenylation element binding protein 2
114801	NM_001258277	TMEM200A	2.939808	6.747186	22.415862	transmembrane protein 200A
7408	NM_003370	VASP	2.930763	50.562700	153.416000	vasodilator-stimulated phosphoprotein
100131315	NR_109767	LOC10013131	2.918706	0.326375	2.787900	uncharacterized LOC100131315
9451	NM_004836	EIF2AK3	2.887992	3.335080	11.543300	eukaryotic translation initiation factor 2-alpha kinase 3
83667	NM_031459	SESN2	2.870872	3.588880	12.241600	sestrin 2
81034	NR_102338,NR	SLC25A32	2.869806	4.563524	15.150020	solute carrier family 25 (mitochondrial folate carrier), member 32
4665	NM_005967	NAB2	2.865593	15.349100	47.432100	NGFI-A binding protein 2 (EGR1 binding protein 2)
7791	NM_001010972	ZYX	2.861074	85.134800	248.535600	zyxin
101928371	NR_110236	AC104134.2	2.860426	1.153480	5.053630	uncharacterized LOC101928371
2247	NM_002006	FGF2	2.839810	4.916720	16.195400	fibroblast growth factor 2 (basic)
22936	NM_012081	ELL2	2.839251	9.029300	28.636700	elongation factor, RNA polymerase II, 2
58515	NM_021237	SELK	2.829372	34.713900	101.229000	selenoprotein K
29775	NM_014550	CARD10	2.804239	4.289770	14.021300	caspase recruitment domain family, member 10
3726	NM_002229	JUNB	2.796692	67.047500	194.375000	jun B proto-oncogene
481	NM_001677	ATP1B1	2.796380	19.563200	59.655800	ATPase, Na+/K+ transporting, beta 1 polypeptide
5205	NM_005603	ATP8B1	2.794569	4.206850	13.717300	ATPase, aminophospholipid transporter, class I, type 8B, member 1
65124	NM_023016	SOWAHC	2.778447	1.746000	6.497760	sosondowah ankyrin repeat domain family member C
84957	NM_152222,NM	RELT	2.758328	0.513443	3.073543	RELT tumor necrosis factor receptor
7057	NM_003246	THBS1	2.755805	96.350000	272.247000	thrombospondin 1
1647	NM_001924,NM	GADD45A	2.747462	56.717960	164.824209	growth arrest and DNA-damage-inducible, alpha
221662	NM_001143941	RBM24	2.746241	5.982274	18.613534	RNA binding motif protein 24
23095	NM_015074,NM	KIF1B	2.742394	5.111290	16.184310	kinesin family member 1B
83855	NM_031918	KLF16	2.737799	11.244700	33.338300	Kruppel-like factor 16
26524	NM_014572	LATS2	2.734320	1.511770	5.768470	large tumor suppressor kinase 2
5366	NM_021127	PMAIP1	2.732568	8.120620	24.773800	phorbol-12-myristate-13-acetate-induced protein 1
255057	NM_152769	C19orf26	2.730839	0.922981	4.151040	chromosome 19 open reading frame 26
644714	NR_033947	LIMD1-AS1	2.709362	4.028390	12.762200	LIMD1 antisense RNA 1
7357	NM_003358	UGCG	2.709170	11.992700	34.870300	UDP-glucose ceramide glucosyltransferase
26511	NM_012110	CHIC2	2.707036	30.834100	86.984400	cysteine-rich hydrophobic domain 2
3875	NM_199187,NM	KRT18	2.703150	228.627900	621.539000	keratin 18
2669	NM_005261,NM	GEM	2.697234	28.859750	82.184800	GTP binding protein overexpressed in skeletal muscle
5468	NM_015869,NM	PPARG	2.687757	6.238874	18.891260	peroxisome proliferator-activated receptor gamma
84275	NM_032315	SLC25A33	2.685006	5.569490	17.033400	solute carrier family 25 (pyrimidine nucleotide carrier), member 33
5277	NR_033835,NR	PIGA	2.680925	0.836676	3.789700	phosphatidylinositol glycan anchor biosynthesis, class A
83874	NM_001204240	TBC1D10A	2.675413	10.790766	31.323628	TBC1 domain family, member 10A
9532	NM_004282	BAG2	2.668440	4.545390	13.969300	BCL2-associated athanogene 2
2303	NM_005251	FOXC2	2.659272	8.408200	24.872600	forkhead box C2 (MFH-1, mesenchyme forkhead 1)
11151	NM_001193333	CORO1A	2.649025	0.331623	2.445310	coronin, actin binding protein, 1A
11153	NM_007076	FICD	2.649004	0.513909	2.925030	FIC domain containing
6144	NM_000982	RPL21	2.638571	42.011800	113.471000	ribosomal protein L21
22856	NM_014918	CHSY1	2.632021	8.367090	24.516700	chondroitin sulfate synthase 1
51665	NM_001040445	ASB1	2.628797	6.668450	19.655600	ankyrin repeat and SOCS box containing 1
23125	NM_001171168	CAMTA2	2.625008	8.482447	24.725657	calmodulin binding transcription activator 2
641467	NR_038381	TSC22D1-AS1	2.612101	0.690790	3.316950	TSC22D1 antisense RNA 1
202915	NM_001097620	TMEM184A	2.609018	2.007930	6.736060	transmembrane protein 184A
6525	NM_001207017	SMTN	2.601422	6.909504	20.162851	smoothelin
90007	NM_177401	MIDN	2.598634	12.275000	34.303400	midnolin
112755	NM_052874	STX1B	2.594218	3.188980	9.950160	syntaxin 1B

9308	NM_004233,NM	CD83	2.594080	0.803399	3.545360	CD83 molecule
10309	NM_021147,NR	CCNO	2.591293	6.773853	19.674350	n/a
9188	NM_004728,NM	DDX21	2.589208	7.358057	21.326797	DEAD (Asp-Glu-Ala-Asp) box helicase 21
3142	NM_021958	HLX	2.580103	1.816150	6.165290	H2.0-like homeobox
148022	NM_182919	TICAM1	2.575413	2.829160	8.928480	toll-like receptor adaptor molecule 1
55132	NM_032239,NM	LARP1B	2.571271	2.937382	9.201886	La ribonucleoprotein domain family, member 1B
339512	NM_178550	C1orf110	2.570965	0.287517	2.225280	chromosome 1 open reading frame 110
126374	NM_00108043€	WTIP	2.567303	7.514390	21.542500	Wilms tumor 1 interacting protein
7424	NM_005429	VEGFC	2.566032	18.998800	52.111600	vascular endothelial growth factor C
23022	NM_00116611C	PALLD	2.563407	19.934703	55.319113	palladin, cytoskeletal associated protein
1850	NM_004420	DUSP8	2.560673	0.038228	1.606300	dual specificity phosphatase 8
2643	NM_00102407C	GCH1	2.559725	0.336648	2.340155	GTP cyclohydrolase 1
490	NM_001682,NM	ATP2B1	2.553061	11.336873	31.485612	ATPase, Ca++ transporting, plasma membrane 1
64859	NM_00103171€	NABP1	2.548768	4.303795	12.687747	nucleic acid binding protein 1
51422	NM_00104063	PRKAG2	2.542550	6.787391	19.325401	protein kinase, AMP-activated, gamma 2 non-catalytic subunit
6876	NM_003186,NM	TAGLN	2.537203	203.417428	524.630780	transgelin
57761	NM_021158,NM	TRIB3	2.528935	9.638433	26.968365	tribbles homolog 3 isoform 1
81929	NM_031216,NM	SEH1L	2.527722	6.436730	18.119160	SEH1-like (S. cerevisiae)
262	NM_001634,NR	AMD1	2.527675	6.941073	19.584917	adenosylmethionine decarboxylase 1
9306	NM_004232	SOCS6	2.522814	5.218540	15.073400	suppressor of cytokine signaling 6
782	NM_199248,NM	CACNB1	2.514614	1.943338	6.299798	calcium channel, voltage-dependent, beta 1 subunit
7430	NM_001111077	EZR	2.510115	55.195200	145.078400	ezrin
8994	NM_014240	LIMD1	2.508692	3.591250	10.569600	LIM domains containing 1
80723	NM_00109759	SLC35G2	2.506351	7.064666	19.758655	solute carrier family 35, member G2
374987	NR_103535	NEXN-AS1	2.506139	0.108868	1.714270	NEXN antisense RNA 1
4154	NM_021038,NM	MBNL1	2.504323	14.228768	38.021528	muscleblind-like splicing regulator 1
4791	NM_002502,NM	NFKB2	2.491396	14.706789	39.014308	nuclear factor of kappa light polypeptide gene enhancer in B-cells 2 (p49/p100)
23135	NM_00108042	KDM6B	2.489819	2.716130	8.275580	lysine (K)-specific demethylase 6B
2114	NM_00125629	ETS2	2.474138	3.369730	9.935134	v-ets avian erythroblastosis virus E26 oncogene homolog 2
11228	NM_00116474	RASSF8	2.473391	11.195781	30.223883	Ras association (RalGDS/AF-6) domain family (N-terminal) member 8
4609	NM_002467	MYC	2.472373	5.859890	16.444600	v-myc avian myelocytomatosis viral oncogene homolog
79850	NM_024792	FAM57A	2.472146	9.872990	26.888400	family with sequence similarity 57, member A
10979	NM_00113499	FERMT2	2.471751	13.204068	34.814190	fermitin family member 2
857	NM_001753,NM	CAV1	2.462569	113.523286	289.584368	caveolin 1, caveolae protein, 22kDa
10560	NM_006996	SLC19A2	2.453579	0.702925	3.062840	solute carrier family 19 (thiamine transporter), member 2
6307	NM_00101736	MSMO1	2.446158	28.766900	74.039200	methylsterol monooxygenase 1
116984	NM_015230	ARAP2	2.445198	0.781272	3.237310	ArfGAP with RhoGAP domain, ankyrin repeat and PH domain 2
858	NM_198212,NM	CAV2	2.443104	11.788805	31.196012	caveolin 2
1264	NM_001299	CNN1	2.443045	0.023379	1.447900	calponin 1, basic, smooth muscle
91283	NM_00119880	MSANTD3	2.439844	24.108732	63.226471	Myb/SANT-like DNA-binding domain containing 3
79693	NM_024640	YRDC	2.439133	7.857860	21.437300	yrdC N(6)-threonylcarbamoyltransferase domain containing
219	NM_000692	ALDH1B1	2.429487	2.385880	7.138700	aldehyde dehydrogenase 1 family, member B1
4664	NM_005966	NAB1	2.426915	6.673240	17.985800	NGFI-A binding protein 1 (EGR1 binding protein 1)
23528	NM_001281294	ZNF281	2.425666	3.294964	9.479729	zinc finger protein 281
51491	NM_00125653	NOP16	2.420676	7.824406	21.222917	nucleolar protein 16 isoform 6
3099	NM_000189	HK2	2.415665	1.058510	3.845580	hexokinase 2
23576	NM_00113444	DDAH1	2.403678	8.461142	22.641980	dimethylarginine dimethylaminohydrolase 1
1263	NM_004073	PLK3	2.402359	12.124800	31.407400	polo-like kinase 3
8394	NM_001135637	PIP5K1A	2.400938	9.916419	26.099372	phosphatidylinositol-4-phosphate 5-kinase, type I, alpha
84230	NM_032270	LRRC8C	2.400525	0.989968	3.632890	leucine rich repeat containing 8 family, member C
134429	NM_139164	STARD4	2.398453	2.380040	7.011930	StAR-related lipid transfer (START) domain containing 4
56342	NM_020230	PPAN	2.398376	8.336820	22.341700	peter pan homolog (Drosophila)
5770	NM_00127861	PTPN1	2.397685	11.009310	28.773000	protein tyrosine phosphatase, non-receptor type 1
84206	NM_032246	MEX3B	2.397437	2.214760	6.614200	mex-3 RNA binding family member B
3727	NM_00128696	JUND	2.395035	119.584000	293.160000	jun D proto-oncogene
401588	NR_015378	ZNF674-AS1	2.389415	1.051620	3.761710	ZNF674 antisense RNA 1 (head to head)
26784	NR_002326	SNORA64	2.386540	6.916020	18.306400	small nucleolar RNA, H/ACA box 64
23223	NM_015179,NM	RRP12	2.377015	9.658006	25.363752	ribosomal RNA processing 12 homolog (S. cerevisiae)
375593	NM_198924	TRIM73	2.374558	0.006017	1.347130	tripartite motif containing 73
1326	NM_001244134	MAP3K8	2.365847	1.044504	3.683990	mitogen-activated protein kinase kinase kinase 8
25816	NM_001077654	TNFAIP8	2.348657	25.867504	64.554756	tumor necrosis factor, alpha-induced protein 8
23082	NM_00128872	PPRC1	2.347376	5.301374	14.118788	peroxisome proliferator-activated receptor gamma, coactivator-related 1
4170	NM_182763,NM	MCL1	2.346029	34.061730	83.040350	myeloid cell leukemia sequence 1 (BCL2-related)
8527	NM_003648,NM	DGKD	2.337288	2.023345	5.982040	diacylglycerol kinase, delta 130kDa
339665	NM_00100147	SLC35E4	2.335065	6.952160	17.971100	solute carrier family 35, member E4
1316	NM_00116012	KLF6	2.330128	8.197529	21.347890	Kruppel-like factor 6
1844	NM_004418	DUSP2	2.327222	0.174704	1.658890	dual specificity phosphatase 2
330	NM_001165,NM	BIRC3	2.321326	0.188775	1.685247	baculoviral IAP repeat containing 3
9262	NM_004226	STK17B	2.318834	4.717690	12.444500	serine/threonine kinase 17b
200894	NM_144996,NM	ARL13B	2.315603	2.249784	6.428414	ADP-ribosylation factor-like 13B
85450	NM_00127201	ITPRIP	2.315551	6.202230	16.146100	inositol 1,4,5-trisphosphate receptor interacting protein
57688	NM_020928	ZSWIM6	2.311194	1.818790	5.426170	zinc finger, SWIM-type containing 6
9943	NM_005109	OXSR1	2.308795	7.550020	19.335400	oxidative stress responsive 1
7803	NM_003463	PTP4A1	2.305595	10.997900	27.484600	protein tyrosine phosphatase type IVA, member 1
22927	NM_014282	HABP4	2.301279	9.514970	24.163200	hyaluronan binding protein 4
116068	NM_198273,NM	LYSMD3	2.290601	4.646793	12.117180	LysM, putative peptidoglycan-binding, domain containing 3
85463	NM_033390	ZC3H12C	2.286757	0.783772	2.967570	zinc finger CCCH-type containing 12C
4603	NM_00108041	MYBL1	2.281089	0.447614	2.197390	myb-related protein A isoform 3
84886	NM_032800,NM	C1orf198	2.280667	5.401005	13.893064	chromosome 1 open reading frame 198
284454	NR_036515	LOC284454	2.274227	11.998700	29.491900	uncharacterized LOC284454
4803	NM_002506	NGF	2.270098	17.120500	40.965000	nerve growth factor (beta polypeptide)
26064	NM_00114552C	RAI14	2.267652	13.667755	33.112296	retinoic acid induced 14
7494	NM_005080,NM	XBP1	2.267411	71.778600	169.902400	X-box binding protein 1
5990	NM_134433,NM	RFX2	2.266026	1.114658	3.637891	regulatory factor X, 2 (influences HLA class II expression)
23064	NM_015046	SETX	2.263784	3.061050	8.223870	senataxin
2182	NM_004458,NM	ACSL4	2.261726	4.797995	12.334470	acyl-CoA synthetase long-chain family member 4
8682	NM_001297577	PEA15	2.257600	29.977604	70.806031	astrocytic phosphoprotein PEA-15 isoform a
90853	NM_001281987	SPOCD1	2.253373	11.120425	27.139519	SPOC domain containing 1
27242	NM_014452	TNFRSF21	2.250579	0.853426	3.057550	tumor necrosis factor receptor superfamily, member 21
5099	NM_002589,NM	PCDH7	2.248151	4.557327	11.614500	protocadherin 7
10611	NM_00101151	PDLIM5	2.246800	49.331854	113.499690	PDZ and LIM domain 5
8915	NM_003921	BCL10	2.246397	8.152300	20.424000	B-cell CLL/lymphoma 10
200424	NM_001287491	TET3	2.244262	1.302730	4.015740	tet methylcytosine dioxygenase 3
8459	NM_003595,NM	TPST2	2.243908	9.220972	22.941219	tyrosylprotein sulfotransferase 2
121512	NM_139241	FGD4	2.241162	0.994664	3.345880	FYVE, RhoGEF and PH domain containing 4
5732	NM_000956	PTGER2	2.238420	0.686019	2.679420	prostaglandin E receptor 2 (subtype EP2), 53kDa
51726	NM_016306	DNAJB11	2.236763	53.138100	122.969000	DnaJ (Hsp40) homolog, subfamily B, member 11
11245	NM_007223,NM	GPR176	2.236697	8.605522	21.403921	G protein-coupled receptor 176
148252	NM_145173	DIRAS1	2.228160	0.632536	2.551010	DIRAS family, GTP-binding RAS-like 1
4953	NM_002539,NM	ODC1	2.227989	84.863606	194.213401	ornithine decarboxylase 1
10950	NM_001130914	BTG3	2.227120	11.358150	27.455100	BTG family, member 3
83593	NM_182664,NM	RASSF5	2.226968	2.017215	5.633892	Ras association (RalGDS/AF-6) domain family member 5
388796	NR_015366,NR	SNHG17	2.225526	7.538170	18.434720	small nucleolar RNA host gene 17 (non-protein coding)
85403	NM_033083	EAF1	2.208586	3.093320	8.064890	ELL associated factor 1
1595	NM_000786,NM	CYP51A1	2.206557	16.085120	37.730370	cytochrome P450, family 51, subfamily A, polypeptide 1
27042	NM_014388	DIEXF	2.201343	1.954100	5.416580	digestive organ expansion factor homolog (zebrafish)
51715	NM_00127866	RAB23	2.194175	2.195318	5.946379	RAB23, member RAS oncogene family
6575	NM_00125718C	SLC20A2	2.187705	5.765252	14.137709	solute carrier family 20 (phosphate transporter), member 2
57205	NM_020453	ATP10D	2.186844	3.487580	8.879650	ATPase, class V, type 10D
100529211	NR_037719	TMEM256-PLS	2.186343	0.805831	2.842860	TMEM256-PLSCR3 readthrough (NMD candidate)
1948	NM_004093	EFNB2	2.182101	2.520070	6.603300	ephrin-B2
29968	NM_021154,NM	PSAT1	2.179447	5.854389	14.281410	phosphoserine aminotransferase 1
27338	NM_014501	UBE2S	2.178090	43.702700	98.486900	ubiquitin-conjugating enzyme E2S
3955	NM_002304,NM	LFNG	2.177302	9.226845	22.291670	LFNG O-fucosylpeptide 3-beta-N-acetylglucosaminyltransferase
10052	NM_00108038	GJC1	2.174853	2.993786	7.641895	gap junction protein, gamma 1, 45kDa
11186	NM_170713,NM	RASSF1	2.168752	6.095088	14.727470	Ras association (RalGDS/AF-6) domain family member 1
55154	NM_00125653	MSTO1	2.168596	6.761359	16.321047	misato 1, mitochondrial distribution and morphology regulator
9645	NM_00128266	MICAL2	2.167490	7.381250	17.558302	microtubule associated monooxygenase, calponin and LIM domain containing 2
9411	NM_004815	ARHGAP29	2.165629	4.119230	10.272500	Rho GTPase activating protein 29
5865	NM_002867	RAB3B	2.162857	1.791520	4.939320	RAB3B, member RAS oncogene family
488	NM_170665,NM	ATP2A2	2.162402	12.679083	29.649401	ATPase, Ca++ transporting, cardiac muscle, slow twitch 2
7185	NM_005658,NM	TRAF1	2.159971	0.640805	2.453480	TNF receptor-associated factor 1

3725	NM_002228	JUN	2.159846	18.614600	42.468700	jun proto-oncogene
2274	NM_001450,NM	FHL2	2.153587	151.252400	325.955309	four and a half LIM domains 2
1130	NM_000081,NR	LYST	2.152867	1.731483	4.760098	lysosomal-trafficking regulator
399694	NM_203349	SHC4	2.151197	0.322523	1.764410	SHC (Src homology 2 domain containing) family, member 4
6197	NM_004586	RPS6KA3	2.149872	3.115690	7.846940	ribosomal protein S6 kinase, 90kDa, polypeptide 3
9253	NM_001289979	NUMBL	2.148591	9.802794	23.244019	numb-like protein isoform b
9456	NM_001277077	HOMER1	2.147713	0.175447	1.453879	homer homolog 1 (Drosophila)
6520	NM_002394,NM	SLC3A2	2.145004	57.050985	128.455193	solute carrier family 3 (amino acid transporter heavy chain), member 2
5817	NM_006505,NM	PVR	2.142668	4.071301	10.019641	poliovirus receptor
54205	NM_018947	CYCS	2.136462	9.493870	22.407200	cytochrome c, somatic
1491	NM_001190463	CTH	2.133516	0.818775	2.769807	cystathionase (cystathionine gamma-lyase)
407975	NR_027350,NR	MIR17HG	2.132680	0.438111	1.970190	miR-17-92 cluster host gene (non-protein coding)
80219	NM_025147	COQ10B	2.132428	12.526800	28.889800	coenzyme Q10 homolog B (S. cerevisiae)
6662	NM_000346	SOX9	2.131754	38.238900	84.039800	SRY (sex determining region Y)-box 9
84919	NM_032833	PPP1R15B	2.126149	4.851600	11.671600	protein phosphatase 1, regulatory subunit 15B
8193	NM_001135156	DPF1	2.123358	0.244494	1.568860	zinc finger protein neuro-d4 isoform d
55917	NM_018704	CTTNBP2NL	2.122611	3.843950	9.315510	CTTNBP2 N-terminal like
5321	NM_024420	PLA2G4A	2.122247	2.712430	6.822750	phospholipase A2, group IVA (cytosolic, calcium-dependent)
7159	NM_001031685	TP53BP2	2.118942	5.571726	13.260194	tumor protein p53 binding protein, 2
84790	NM_001303115	TUBA1C	2.115575	87.774044	191.454006	tubulin, alpha 1c
100505854	NR_038361	RSBN1L-AS1	2.114853	4.001850	9.721420	RSBN1L antisense RNA 1
55361	NM_018425	PI4K2A	2.106969	12.058300	27.365900	phosphatidylinositol 4-kinase type 2 alpha
219333	NM_182488	USP12	2.105217	5.914610	13.891100	ubiquitin specific peptidase 12
100134259	NR_024452	LINC01119	2.099893	1.341440	3.751790	long intergenic non-protein coding RNA 1119
79929	NM_024871	MAP6D1	2.099777	0.603998	2.268490	MAP6 domain containing 1
27161	NM_001164623	AGO2	2.099243	0.899852	2.881486	argonaute RISC catalytic component 2
10678	NM_006577	B3GNT2	2.098008	1.879040	4.936840	UDP-GlcNAc:betaGal beta-1,3-N-acetylglucosaminyltransferase 2
150142	NR_027273,NR	ZNF295-AS1	2.097127	1.076204	3.225834	n/a
402682	NM_001015072	UFSP1	2.093759	1.896300	4.963740	UFM1-specific peptidase 1 (non-functional)
8660	NM_003749	IRS2	2.090274	5.092020	11.992700	insulin receptor substrate 2
5074	NM_002583	PAWR	2.089592	7.420940	16.989500	PRKC, apoptosis, WT1, regulator
10383	NM_006088	TUBB4B	2.081751	73.772800	158.182000	tubulin, beta 4B class IVb
221079	NM_178815	ARL5B	2.081372	0.625954	2.292430	ADP-ribosylation factor-like 5B
100302652	NM_001164165	GPR75-ASB3	2.081138	0.000046	1.041970	GPR75-ASB3 readthrough
11010	NM_006851	GLIPR1	2.081063	12.515800	28.150800	GLI pathogenesis-related 1
57089	NM_020354	ENTPD7	2.080565	2.013740	5.174800	ectonucleoside triphosphate diphosphohydrolase 7
25897	NM_183419,NM	RNF19A	2.079385	4.071094	9.673152	ring finger protein 19A, RBR E3 ubiquitin protein ligase
11040	NM_006875	PIM2	2.079102	5.221220	12.247400	pim-2 oncogene
84447	NM_032431,NM	SYVN1	2.077690	14.906900	32.970400	synovial apoptosis inhibitor 1, synoviolin
283209	NM_173582	PGM2L1	2.075790	0.764984	2.560500	phosphoglucomutase 2-like 1
8654	NM_033437,NM	PDE5A	2.075374	1.951343	5.009377	phosphodiesterase 5A, cGMP-specific
3484	NM_000596	IGFBP1	2.071323	1.676930	4.401680	insulin-like growth factor binding protein 1
10458	NM_001144888	BAIAP2	2.069391	19.401059	42.577103	BAI1-associated protein 2
4208	NM_001193347	MEF2C	2.066599	0.922819	2.869231	myocyte enhancer factor 2C
50814	NM_015922,NM	NSDHL	2.054548	12.347026	27.378566	NAD(P) dependent steroid dehydrogenase-like
23002	NM_001270520	DAAM1	2.050389	3.053721	7.278330	dishevelled associated activator of morphogenesis 1
401093	NR_027038,NR	MBNL1-AS1	2.047501	0.339500	1.662655	MBNL1 antisense RNA 1
387496	NM_206827	RASL11A	2.046961	2.363590	5.804380	RAS-like, family 11, member A
6478	NM_005067	SIAH2	2.046513	6.729630	15.171800	siah E3 ubiquitin protein ligase 2
283638	NM_015005,NM	CEP170B	2.043472	4.835571	11.112610	centrosomal protein 170B
7262	NM_003311	PHLDA2	2.042800	56.480200	119.957000	pleckstrin homology-like domain, family A, member 2
55201	NM_018174	MAP1S	2.042710	7.783790	17.483000	microtubule-associated protein 1S
4839	NM_001258308	NOP2	2.038070	7.453727	16.658327	NOP2 nucleolar protein
3182	NM_031266,NM	HNRNPAB	2.036936	30.162500	64.660200	heterogeneous nuclear ribonucleoprotein A/B
9957	NM_005114	HS3ST1	2.030168	6.607970	14.756800	heparan sulfate (glucosamine) 3-O-sulfotransferase 1
10602	NM_006449,NM	CDC42EP3	2.028410	15.348140	33.233856	CDC42 effector protein (Rho GTPase binding) 3
10150	NM_144778,NM	MBNL2	2.026813	6.611641	14.744910	muscleblind-like splicing regulator 2
9589	NM_004906,NM	WTAP	2.026235	51.625632	107.385798	Wilms tumor 1 associated protein
57835	NM_021196,NM	SLC4A5	2.025762	0.478828	1.900370	solute carrier family 4 (sodium bicarbonate cotransporter), member 5
26278	NM_001278055	SACS	2.024639	1.144871	3.205431	spastic ataxia of Charlevoix-Saguenay (sacsin)
84159	NM_001244638	ARID5B	2.015813	6.136094	13.677961	AT rich interactive domain 5B (MRF1-like)
541471	NR_024373,NR	MIR4435-1HG	2.009149	81.118923	170.616678	MIR4435-1 host gene (non-protein coding)
91694	NM_152271	LONRF1	2.006265	1.379120	3.613210	LON peptidase N-terminal domain and ring finger 1
100874207	NR_046683	ST3GAL6-AS1	2.004240	2.574970	6.126670	ST3GAL6 antisense RNA 1
26024	NM_015545	PTCD1	−2.005381	1.041030	0.000788	pentatricopeptide repeat domain 1
84273	NM_032313	NOA1	−2.012689	21.354000	10.540800	nitric oxide associated 1
155382	NM_001077621	VPS37D	−2.014338	10.864400	4.977530	vacuolar protein sorting 37 homolog D (S. cerevisiae)
60686	NM_001130706	C14orf93	−2.027676	5.632010	2.230865	chromosome 14 open reading frame 93
5900	NM_001271776	RALGDS	−2.029608	22.120842	10.824013	ral guanine nucleotide dissociation stimulator
219539	NM_145008	YPEL4	−2.040006	4.305230	1.534110	yippee-like 4 (Drosophila)
8718	NM_148970,NM	TNFRSF25	−2.043808	18.745381	9.039218	tumor necrosis factor receptor superfamily, member 25
25988	NM_198971,NM	HINFP	−2.056788	7.702937	3.238709	histone H4 transcription factor
51545	NM_016535	ZNF581	−2.058803	44.594700	21.981800	zinc finger protein 581
375387	NM_198565	NRROS	−2.065625	2.975550	0.874285	negative regulator of reactive oxygen species
10252	NM_001258039	SPRY1	−2.067407	3.358284	1.045718	sprouty homolog 1, antagonist of FGF signaling (Drosophila)
860	NM_001278478	RUNX2	−2.069360	6.209299	2.453975	runt-related transcription factor 2
79365	NM_030762	BHLHE41	−2.071126	60.849100	30.240400	basic helix-loop-helix family, member e41
388115	NM_207380	C15orf52	−2.074918	21.367100	10.267200	chromosome 15 open reading frame 52
55196	NM_018169	KIAA1551	−2.075862	3.925780	1.309340	KIAA1551
9249	NM_004753	DHRS3	−2.075956	58.295800	28.895200	dehydrogenase/reductase (SDR family) member 3
93129	NM_152288	ORAI3	−2.079228	32.735000	15.666300	ORAI calcium release-activated calcium modulator 3
286827	NM_173084	TRIM59	−2.081590	4.463590	1.557200	tripartite motif containing 59
7582	NM_006955	ZNF33B	−2.086414	4.082700	1.367900	zinc finger protein 33B
54838	NM_017787,NM	WBP1L	−2.086945	26.008487	12.367080	WW domain binding protein 1-like
157638	NM_174911	FAM84B	−2.100512	3.491710	1.071050	family with sequence similarity 84, member B
994	NM_001287522	CDC25B	−2.114881	14.222691	6.341163	cell division cycle 25B
375287	NM_198557	RBM43	−2.141285	2.893790	0.770185	RNA binding motif protein 43
79845	NM_024787	RNF122	−2.145252	1.980850	0.349732	ring finger protein 122
2074	NM_000124	ERCC6	−2.151088	7.990810	3.188860	excision repair cross-complementing rodent repair deficiency, complementation group 6
1912	NM_198040,NM	PHC2	−2.157255	86.964406	40.408662	polyhomeotic homolog 2 (Drosophila)
84674	NM_032587	CARD6	−2.158480	3.653750	1.086370	caspase recruitment domain family, member 6
4081	NM_005584	MAB21L1	−2.160748	55.666300	26.340400	mab-21-like 1 (C. elegans)
54873	NM_017734	PALMD	−2.174277	8.956730	3.598170	palmdelphin
3570	NM_181359,NM	IL6R	−2.184810	2.991252	0.774959	interleukin 6 receptor
3162	NM_002133	HMOX1	−2.198589	20.351900	9.128710	heme oxygenase (decycling) 1
677805	NR_002959	SNORA18	−2.238620	31.074500	13.709100	small nucleolar RNA, H/ACA box 18
9595	NM_004288	CYTIP	−2.244024	3.559980	0.972638	cytohesin 1 interacting protein
149076	NM_152493	ZNF362	−2.244113	13.196300	5.451710	zinc finger protein 362
654320	NR_002920	SNORA8	−2.249914	31.754600	14.006700	small nucleolar RNA, H/ACA box 8
131583	NM_153690	FAM43A	−2.260313	1.838220	0.222324	family with sequence similarity 43, member A
23179	NM_001297672	RGL1	−2.263622	13.730300	5.650239	ral guanine nucleotide dissociation stimulator-like 1 isoform 3
79927	NM_024869	FAM110D	−2.263748	1.785580	0.195380	family with sequence similarity 110, member D
55729	NM_001286515	ATF7IP	−2.273111	6.679400	2.348219	activating transcription factor 7 interacting protein
388886	NM_207644	FAM211B	−2.281566	10.209700	3.955650	family with sequence similarity 211, member B
677768	NR_003002	SCARNA13	−2.282071	1.332830	0.000281	small Cajal body-specific RNA 13
23401	NM_012083	FRAT2	−2.282654	7.316020	2.616330	frequently rearranged in advanced T-cell lymphomas 2
8111	NM_001177676	GPR68	−2.285864	2.542909	0.512811	G protein-coupled receptor 68
7133	NM_001066	TNFRSF1B	−2.286504	3.597480	0.952975	tumor necrosis factor receptor superfamily, member 1B
85027	NM_032947	SMIM3	−2.291433	50.672000	22.342000	small integral membrane protein 3
267004	NM_170753	PGBD3	−2.298951	28.238700	12.152900	piggyBac transposable element derived 3
63939	NM_022106,NM	FAM217B	−2.301705	2.635306	0.539404	family with sequence similarity 217, member B
79899	NM_024841,NM	PRR5L	−2.311599	6.620821	2.254408	proline rich 5 like
23338	NM_001289985	JADE2	−2.319340	4.743007	1.421715	protein Jade-2 isoform 1
57172	NM_020439	CAMK1G	−2.330019	4.732880	1.409070	calcium/calmodulin-dependent protein kinase IG
84687	NM_032595	PPP1R9B	−2.341296	40.330000	17.034100	protein phosphatase 1, regulatory subunit 9B
1628	NM_001352	DBP	−2.361454	12.310300	4.735650	D site of albumin promoter (albumin D-box) binding protein
56997	NM_020247	ADCK3	−2.363703	8.268840	2.942010	aarF domain containing kinase 3
54899	NM_001289095	PXK	−2.404350	24.201768	9.905354	PX domain containing serine/threonine kinase
359948	NM_001077397	IRF2BP2	−2.417404	22.639940	9.158460	interferon regulatory factor 2 binding protein 2
219654	NM_153367	ZCCHC24	−2.444526	24.876200	10.009700	zinc finger, CCHC domain containing 24
94241	NM_001135733	TP53INP1	−2.460345	13.256438	4.896366	tumor protein p53 inducible nuclear protein 1
652	NM_130850,NM	BMP4	−2.487004	29.620240	11.720784	bone morphogenetic protein 4

10614	NM_006460	HEXIM1	**−2.490046**	34.966600	13.773200	hexamethylene bis−acetamide inducible 1
389119	NM_203370	FAM212A	**−2.490971**	3.100280	0.608098	family with sequence similarity 212, member A
55812	NM_001040428	SPATA7	**−2.491040**	10.531980	3.633783	spermatogenesis associated 7
3037	NM_005328	HAS2	**−2.498860**	2.800520	0.489938	hyaluronan synthase 2
1831	NM_001015881	TSC22D3	**−2.516391**	82.533440	32.965636	TSC22 domain family, member 3
10826	NM_032385	FAXDC2	**−2.519795**	9.045100	3.018120	fatty acid hydroxylase domain containing 2
100532735	NR_037849	INO80B−WBP1	**−2.523961**	1.575360	0.000184	INO80B−WBP1 readthrough (NMD candidate)
124790	NM_144608,NM	HEXIM2	**−2.532373**	13.255914	4.703251	hexamethylene bis−acetamide inducible 2
10586	NM_006439	MAB21L2	**−2.566357**	102.155000	39.496200	mab−21−like 2 (C. elegans)
8651	NM_003745	SOCS1	**−2.586432**	7.144540	2.118030	suppressor of cytokine signaling 1
284358	NM_001130915	MAMSTR	**−2.597470**	4.031128	0.890481	MEF2−activating motif and SAP domain−containing transcriptional regulator isoform 3
200845	NM_153331,NM	KCTD6	**−2.599423**	19.843465	7.287366	potassium channel tetramerization domain containing 6
9021	NM_003955	SOCS3	**−2.612614**	8.964190	2.847120	suppressor of cytokine signaling 3
84722	NM_001005290	PSRC1	**−2.634602**	5.493490	1.424811	proline/serine−rich coiled−coil 1
2535	NM_001466	FZD2	**−2.638606**	20.757100	7.495160	frizzled family receptor 2
57561	NM_020801	ARRDC3	**−2.646088**	16.680300	5.841960	arrestin domain containing 3
23580	NM_012121	CDC42EP4	**−2.689530**	9.992900	3.102850	CDC42 effector protein (Rho GTPase binding) 4
9572	NM_021724	NR1D1	**−2.732584**	4.909910	1.117160	nuclear receptor subfamily 1, group D, member 1
7023	NM_003223	TFAP4	**−2.758183**	2.916340	0.386092	transcription factor AP−4 (activating enhancer binding protein 4)
7056	NM_000361	THBD	**−2.818718**	18.798800	6.208350	thrombomodulin
3437	NM_001289758	IFIT3	**−2.843869**	4.574415	0.918419	interferon−induced protein with tetratricopeptide repeats 3 isoform b
6297	NR_111966,NR	SALL2	**−2.851992**	10.329752	2.984687	sal−like protein 2 isoform b
10023	NM_005479	FRAT1	**−2.939676**	3.747840	0.573165	frequently rearranged in advanced T−cell lymphomas
6495	NM_005982	SIX1	**−2.970255**	39.120500	12.836600	SIX homeobox 1
10276	NM_001047160	NET1	**−2.976787**	53.186630	17.863959	neuroepithelial cell transforming 1
8092	NM_006982	ALX1	**−3.111645**	3.871140	0.530015	ALX homeobox 1
51063	NR_024552,NM	CALHM2	**−3.183028**	19.294790	5.565420	calcium homeostasis modulator 2
54541	NM_019058	DDIT4	**−3.197649**	145.565000	45.882400	DNA−damage−inducible transcript 4
7090	NM_001105192	TLE3	**−3.243055**	19.418781	5.491108	transducin−like enhancer of split 3 (E(sp1) homolog, Drosophila)
677822	NR_002973	SNORA40	**−3.273466**	32.003600	9.412180	small nucleolar RNA, H/ACA box 40
101929351	NR_121597,NR	CDC37L1−AS1	**−3.277680**	18.332450	5.012821	n/a
6659	NM_003107	SOX4	**−3.355183**	29.649100	8.443500	SRY (sex determining region Y)−box 4
27244	NM_001199933	SESN1	**−3.393443**	12.165501	2.897486	sestrin 1
2307	NM_004118	FOXS1	**−3.415744**	18.062300	4.666280	forkhead box S1
1846	NM_057158,NM	DUSP4	**−3.484403**	74.494217	21.363978	dual specificity phosphatase 4
8503	NM_001303427	PIK3R3	**−3.534229**	15.541640	3.725776	phosphoinositide−3−kinase, regulatory subunit 3 (gamma)
2297	NM_004472	FOXD1	**−3.665924**	118.585000	32.202900	forkhead box D1
113791	NM_001135911	PIK3IP1	**−3.702383**	20.915390	5.079671	phosphoinositide−3−kinase interacting protein 1
3397	NM_181353,NM	ID1	**−3.794072**	146.374590	38.412880	inhibitor of DNA binding 1, dominant negative helix−loop−helix protein
10018	NM_138622,NM	BCL2L11	**−3.828162**	9.005039	1.613471	BCL2−like 11 (apoptosis facilitator)
5507	NM_005398	PPP1R3C	**−3.873820**	13.531800	2.766170	protein phosphatase 1, regulatory subunit 3C
170689	NM_139055	ADAMTS15	**−3.931771**	7.249030	1.069160	ADAM metallopeptidase with thrombospondin type 1 motif, 15
27113	NM_001127241	BBC3	**−3.977221**	12.734935	2.466995	BCL2 binding component 3
3398	NM_002166	ID2	**−4.192023**	8.887690	1.357040	inhibitor of DNA binding 2, dominant negative helix−loop−helix protein
3433	NM_001547	IFIT2	**−4.201360**	6.021260	0.656446	interferon−induced protein with tetratricopeptide repeats 2
64207	NM_024496	IRF2BPL	**−4.263684**	51.579700	11.778600	interferon regulatory factor 2 binding protein−like
84913	NM_032827	ATOH8	**−4.297373**	13.154600	2.294630	atonal homolog 8 (Drosophila)
387914	NM_001007538	SHISA2	**−4.533360**	53.224500	11.420800	shisa family member 2
602	NM_005178	BCL3	**−4.578218**	49.932600	10.463400	B−cell CLL/lymphoma 3
5015	NM_001270523	OTX2	**−4.649469**	25.725919	4.858800	orthodenticle homeobox 2
1027	NM_004064	CDKN1B	**−4.766649**	115.986000	24.212100	cyclin−dependent kinase inhibitor 1B (p27, Kip1)
8325	NM_031866	FZD8	**−7.338992**	44.399700	5.307110	frizzled family receptor 8
90427	NM_001003943	BMF	**−14.362669**	49.725362	2.524667	Bcl2 modifying factor
10628	NM_006472	TXNIP	**−20.335814**	123.344000	5.160960	thioredoxin interacting protein
