# Supplementary material for: Ciliary GPCR‐based transcriptome as a key regulator of cilia length control
Source: FASEB Bioadv. 2021 Jul 5;3(9):744–67. doi: 10.1096/fba.2021-00029 (PMC8409570; doi:10.1096/fba.2021-00029)
Supplement: Supplementary file 7 — Table S6 [file FBA2-3-744-s007.pdf]

Supplemental Table 6. The *p*-value of Fig.2 (relative gene expression levels of MCHR1:EGFP clone cells treated with MCH)

Gene name	MCH 0 hr v.s. 0.5 hr	MCH 0 hr v.s. 2 hr	MCH 0 hr v.s. 4 hr
ATF3	$p < 0.001$	$p < 0.001$	n.s.
ARC	n.s.	$p < 0.001$	n.s.
BMF	n.s.	$p = 0.0037$	$p = 0.015$
FOSB	$p < 0.001$	$p < 0.001$	$p = 0.0016$
MAFF	$p = 0.012$	n.s.	n.s.
PDLIM5	$p = 0.0031$	$p < 0.001$	n.s.
PRKAG2	n.s.	$p = 0.0041$	n.s.
RAB3B	n.s.	$p < 0.001$	$p < 0.001$
RAB23	n.s.	$p = 0.0025$	n.s.
RGS2	$p < 0.001$	$p < 0.001$	$p < 0.001$
RGS3	$p = 0.0018$	$p < 0.001$	$p < 0.001$
RGS4	$p = 0.0031$	$p = 0.0017$	$p = 0.0028$

Significant differences relative to control are determined using the Tukey–Kramer method.　n.s.: not significant
