# Supplementary material for: Ciliary GPCR‐based transcriptome as a key regulator of cilia length control
Source: FASEB Bioadv. 2021 Jul 5;3(9):744–67. doi: 10.1096/fba.2021-00029 (PMC8409570; doi:10.1096/fba.2021-00029)
Supplement: Supplementary file 8 — Table S7 [file FBA2-3-744-s001.pdf]

Supplemental Table 7. The *p*-value of Fig.3 (effect of PTX, Akti1/2, and SP600125 on each gene fluctuated after MCH treatment)

Gene name	No treatment	3 ng/ml PTX	10 ng/ml PTX	3 µM Akti1/2 +3 µM SP600125	10 µM Akti1/2 +10 µM SP600125
ATF3	$p < 0.001$	n.s.	n.s.	$p < 0.001$	$p < 0.001$
ARC	$p < 0.001$	$p < 0.001$	$p < 0.001$	$p < 0.001$	$p < 0.001$
BMF	$p < 0.001$	n.s.	$p = 0.0082$	$p = 0.0036$	$p = 0.0026$
FOSB	$p < 0.001$	n.s.	n.s.	$p < 0.001$	$p < 0.001$
MAFF	n.s.	n.s.	n.s.	n.s.	n.s.
PDLIM5	$p < 0.001$	n.s.	n.s.	$p = 0.021$	n.s.
PRKAG2	$p < 0.001$	$p = 0.0021$	$p = 0.0031$	$p < 0.001$	$p < 0.001$
RAB3B	$p < 0.001$	$p < 0.001$	$p = 0.0041$	$p = 0.0026$	$p < 0.001$
RAB23	$p = 0.0039$	$p = 0.0032$	$p < 0.001$	$p < 0.001$	$p = 0.0027$
RGS2	$p < 0.001$	$p < 0.001$	$p < 0.001$	$p < 0.001$	$p < 0.001$
RGS3	$p < 0.001$	n.s.	n.s.	$p = 0.017$	n.s.
RGS4	$p < 0.001$	$p < 0.001$	$p < 0.001$	$p < 0.001$	$p < 0.001$

Significant differences relative to control are determined using the Tukey–Kramer method.　n.s.: not significant
